# The Added Benefit of Intra‐Arterial Thrombolysis After Successful Recanalization by Endovascular Treatment: A Systematic Review and Meta‐Analysis of Randomized‐Controlled Clinical Trials

**DOI:** 10.1111/ene.70270

**Published:** 2025-07-07

**Authors:** Lina Palaiodimou, Nikolaos M. Papageorgiou, Guillaume Turc, Benjamin Gory, Aikaterini Theodorou, Eleni Bakola, George Magoufis, Stavros Spiliopoulos, Michail Mantatzis, Nitin Goyal, Marios Themistocleous, Amrou Sarraj, Aristeidis H. Katsanos, Urs Fischer, Andrei V. Alexandrov, Georgios Tsivgoulis

**Affiliations:** ^1^ Second Department of Neurology, ‘Attikon’ University Hospital, School of Medicine National & Kapodistrian University of Athens Athens Greece; ^2^ Institute of Psychiatry and Neuroscience of Paris (IPNP), INSERM U1266 Université Paris Cité Paris France; ^3^ Department of Neurology GHU‐Paris Psychiatrie et Neurosciences Paris France; ^4^ CHRU‐Nancy, Department of Diagnostic and Therapeutic Neuroradiology Université de Lorraine Nancy France; ^5^ CHRU‐Nancy, INSERM U1254 Université de Lorraine, CIC, Innovations Technologiques Nancy France; ^6^ Interventional Neuroradiology Unit Metropolitan Hospital Piraeus Greece; ^7^ Interventional Radiology Unit, Second Department of Radiology ‘Attikon’ University General Hospital, National & Kapodistrian University of Athens Athens Greece; ^8^ Radiology Department of University Hospital of Thessaloniki, AHEPA Aristotle University of Thessaloniki, University Campus Thessaloniki Greece; ^9^ Department of Neurology University of Tennessee Health Sciences Center Memphis Tennessee USA; ^10^ Neurosurgery Semmes Murphey Foundation Memphis Tennessee USA; ^11^ Neurosurgical Department Pediatric Hospital of Athens, ‘Agia Sophia’ Athens Greece; ^12^ Department of Neurology University Hospitals Cleveland Medical Center Cleveland Ohio USA; ^13^ Division of Neurology McMaster University and Population Health Research Institute Hamilton Ontario Canada; ^14^ Department of Neurology Inselspital Bern University Hospital, University of Bern Bern Switzerland; ^15^ Department of Neurology Banner University Hospital, University of Arizona College of Medicine Phoenix USA

**Keywords:** acute ischemic stroke, endovascular treatment, intra‐arterial thrombolysis, meta‐analysis, randomized‐controlled clinical trial, successful recanalization, systematic review

## Abstract

**Background:**

Despite successful recanalization following endovascular thrombectomy (EVT) for acute ischemic stroke (AIS) with large‐vessel occlusion (LVO), many patients fail to achieve excellent functional outcomes. Post‐EVT intra‐arterial thrombolysis (IAT) has emerged as a potential adjunctive strategy to improve microvascular reperfusion and clinical recovery.

**Methods:**

We conducted a systematic review and meta‐analysis of randomized‐controlled clinical trials (RCTs) comparing IAT plus best medical therapy (BMT) versus BMT alone in LVO‐AIS patients with successful recanalization post‐EVT. The primary efficacy outcome was 3‐month excellent functional outcome [modified Rankin Scale (mRS)‐score: 0–1]. Secondary efficacy outcomes included good functional outcome (mRS‐score: 0–2) and reduced disability (mRS‐score shift analysis) at 3 months. The primary safety outcome was symptomatic intracranial hemorrhage (sICH); secondary safety outcomes included any‐ICH and 3‐month all‐cause mortality. Subgroup and network meta‐analyses were performed evaluating the effects of different thrombolytic agents.

**Results:**

Seven RCTs were included, comprising 1083 patients treated with IAT and 1048 patients treated with BMT alone. IAT was associated with higher likelihood of excellent functional outcome (RR: 1.23; 95% CI: 1.11–1.36; *I* ^2^ = 0%) and reduced disability at 3 months (common‐OR: 1.10; 95% CI: 1.03–1.18; *I* ^2^ = 0%) compared with BMT alone. Similar rates of 3‐month good functional outcome, 3‐month mortality, sICH and any‐ICH were observed. Although no significant subgroup differences emerged, in the network meta‐analysis alteplase ranked highest in efficacy [surface under the cumulative rank curve (SUCRA): 90%], followed by tenecteplase (61%) and urokinase (40%) in achieving 3‐month excellent functional outcome.

**Conclusions:**

IAT improves excellent functional outcomes without compromising safety in LVO‐AIS patients with successful recanalization after EVT.

**Trial Registration:**

The prespecified protocol of the present systematic review and meta‐analysis has been registered in the International Prospective Register of Ongoing Systematic Reviews PROSPERO (registration ID: CRD420251035903)

## 1 Introduction

Acute ischemic stroke (AIS) due to large‐vessel occlusion (LVO) remains a leading cause of long‐term disability and mortality worldwide [1]. Over the past decade, endovascular treatment (EVT) has transformed the therapeutic landscape for these patients, with randomized‐controlled clinical trials (RCTs) establishing EVT as the standard of care for anterior circulation LVOs and basilar artery occlusion [2, 3, 4]. Despite this breakthrough, functional independence is not achieved in nearly half of AIS patients with LVO, even when successful recanalization [defined as an expanded Thrombolysis in Cerebral Infarction (eTICI) score of 2b‐3] is obtained [5, 6]. This discrepancy between angiographic success and clinical outcomes has underscored a critical limitation: successful recanalization does not always equate to successful reperfusion. An estimated 30%–50% of patients experience persistent microvascular hypoperfusion postthrombectomy, attributed to mechanisms such as the “no‐reflow” phenomenon, microthromboembolism, and capillary dysfunction [7, 8, 9]. This incomplete tissue‐level reperfusion is increasingly recognized as a key determinant of poor prognosis, even in technically successful procedures.

Intra‐arterial thrombolysis (IAT), first explored in early stroke trials as a sole treatment [10], has recently regained attention as a potential adjunct to EVT. By targeting residual thrombi in the distal vasculature, IAT may enhance microcirculatory perfusion and limit infarct expansion [9, 11, 12]. Importantly, localized administration of thrombolytics offers the theoretical advantage of maximizing efficacy while minimizing systemic bleeding risk. Several recent RCTs have assessed IAT in patients who achieved successful recanalization following EVT, yet their findings have been inconsistent. While some trials have demonstrated improvements in functional outcomes, others have reported neutral effects, raising questions about the optimal use of IAT, the most effective thrombolytic agents, and patient subgroups most likely to benefit.

In this context, we conducted a systematic review and pairwise meta‐analysis of RCTs to evaluate the efficacy and safety of adjunctive IAT combined with best medical therapy (BMT) compared to BMT alone in adult patients with AIS due to LVO who achieved successful recanalization post‐EVT. By integrating data from the most recent high‐quality trials, this study aims to clarify the role of IAT in modern stroke care and inform evidence‐based clinical decision‐making.

## 2 Methods

### 2.1 Standard Protocol Approvals, Registrations, and Patient Consents

The prespecified protocol of the present systematic review and meta‐analysis has been registered in the International Prospective Register of Ongoing Systematic Reviews PROSPERO (registration ID: CRD420251035903; date of registration 18 Apr 2025). The meta‐analysis is reported according to the updated Preferred Reporting Items for Systematic Reviews and Meta‐Analyses (PRISMA) guidelines [13]. This study did not require an ethical board approval or written informed consent by the patients according to the study design (systematic review and meta‐analysis).

### 2.2 Data Sources, Searches and Study Selection

A systematic literature search was conducted according to the patient, intervention, comparison, and outcome (PICO) model [14] to identify available RCTs including adult patients with successful recanalization post‐EVT (P: population) receiving IAT together with BMT (I: intervention) versus BMT alone with or without placebo (C: control) and investigating the outcomes of interest as outlined below (O: outcome). Successful recanalization was defined as an eTICI score of 2b, 2c, or 3 post‐EVT [2, 3]. EVT was administered in both groups and comprised the use of stent retrievers, thrombaspiration, balloon angioplasty, stent deployment, or a combination of these approaches. BMT was defined as standard care, which could include preadministration of intravenous thrombolysis (IVT) before EVT.

Observational cohort studies, noncontrolled studies, case series and case reports were excluded. Studies that included IAT‐treated patients without successful recanalization post‐EVT were excluded. Commentaries, editorials, and narrative reviews were also excluded.

The literature search was performed independently by four reviewers (L.P., N.M.P., A.T., and E.B.). The electronic databases MEDLINE and Scopus were searched, using search strings that included the terms “intra‐arterial thrombolysis”, “endovascular treatment” and “successful recanalization”. The complete search algorithm is provided in the Supplement. No language or other restrictions were applied. Our search spanned from the inception of each database to April 20th, 2025. Furthermore, the reference lists of published articles and international conference abstracts were manually scrutinized to ensure the completeness of the bibliography. All retrieved studies were independently assessed by four reviewers (L.P., N.M.P., A.T., and E.B.) and any disagreements were resolved after discussion with a fifth tie‐breaking evaluator (G.T.s).

### 2.3 Quality Control, Bias Assessment and Data Extraction

Four reviewers (L.P., N.M.P., A.T., and E.B.) independently assessed quality control and bias assessment among eligible studies, employing the Cochrane Collaboration Risk‐of‐Bias 2 tool (RoB 2) for RCTs [15]. Any disagreements were settled by consensus after discussion with the corresponding author (GTs).

Data extraction was performed in structured reports, including study name, country, recruitment period, intervention and comparison characteristics, included patients, their baseline characteristics, and the outcomes of interest.

### 2.4 Outcomes

The primary efficacy outcome of interest was excellent functional outcome at 3 months, defined as the modified Rankin Scale (mRS) score ≤ 1 [16]. Good functional outcome at 3 months (defined as mRS ≤ 2) [16], and reduced disability at 3 months (defined as at least 1‐point reduction across all mRS strata) [12] were assessed as secondary efficacy endpoints.

The primary safety outcome was symptomatic intracranial hemorrhage (sICH) as defined in each individual RCT. Any intracranial hemorrhage (any‐ICH) and all‐cause mortality at 3 months were assessed as secondary safety outcomes.

### 2.5 Statistical Analysis

For the pairwise meta‐analysis, we calculated risk ratios (RR) with 95% confidence intervals (CIs) for dichotomous outcomes—that is, the comparison of outcome events among patients receiving IAT versus BMT. For every outcome of interest, the corresponding pooled proportion with 95% CI was calculated for each arm, after the implementation of the variance‐stabilizing double arcsine transformation. For the evaluation of reduced disability at 3 months, the unadjusted common odds ratio (cOR) with 95% CI was calculated using generic inverse variance meta‐analysis. Prespecified subgroup analysis was conducted stratified by the thrombolytic agent used (alteplase; tenecteplase; urokinase). All outcomes were assessed based on intention‐to‐treat analysis. For the primary efficacy outcome, the number needed to treat (NNT) was calculated using the formula: $\mathrm{NNT}=\frac{1}{\left(\mathrm{RR}-1\right)\;x\;\text{rate in}\;\mathrm{BMT}\;\text{group}}$ as previously described [17]. A sensitivity analysis was further conducted for the primary efficacy endpoint, stratified by country of recruitment in each trial. Treatment effect modifications by IVT pretreatment, relevant dose of thrombolytic drug (% licensed full‐dose for IVT), or degree of reperfusion (eTICI 2b vs. eTICI 2c/3) for the primary efficacy endpoint were tested through subgroup analyses. Comparison of the baseline characteristics to assess the balance between the two arms was performed using odds ratios for dichotomous variables and standardized mean differences for continuous variables. For studies reporting continuous outcomes in median values and corresponding interquartile ranges, we estimated the sample mean and standard deviation using the quantile estimation method [18]. The random‐effects model (DerSimonian and Laird 1986) was used to calculate the pooled estimates [19].

The threshold for statistical significance for the above analyses was set at two‐sided *p*‐value of < 0.05. Heterogeneity was assessed with the *I* ^2^ and Cochran *Q* test. For the qualitative interpretation of heterogeneity, *I* ^2^ values <25%, between 25%–50% and > 50% were considered to represent low, moderate, and significant heterogeneity, respectively. The significance level for the *Q* statistic was set at < 0.1. Small‐study effects, as a proxy for publication bias, were assessed when at least four studies were included in the analysis of the outcomes of interest, using funnel plot inspection. Prediction intervals (PI) were also calculated for all outcomes of interest to estimate the range of effects in future studies, as previously performed by our group [20]. The above statistical analyses were performed using the R software version 3.5.0 (package: meta) [21].

Finally, a frequentist random‐effect network meta‐analysis (NMA) was conducted for the primary efficacy outcome of interest. The principal summary measure was the RR and its respective 95% CI, estimated by arm‐based analyses. Secondary measures of treatment effects were calculated for each thrombolytic agent with surface under the cumulative rank curve (SUCRA) probabilities and treatment rankings. All results were presented with higher SUCRA values indicating better performance (i.e., higher rate of achieving an excellent functional outcome at 3 months). Inconsistency tests were conducted with the node‐split model method to assess whether the direct and indirect evidence is consistent, with the significance level set at *p* < 0.05. NMA was conducted using the MetaInsight web‐based tool [22].

## 3 Results

### 3.1 Literature Search and Included Studies

**FIGURE 1:**
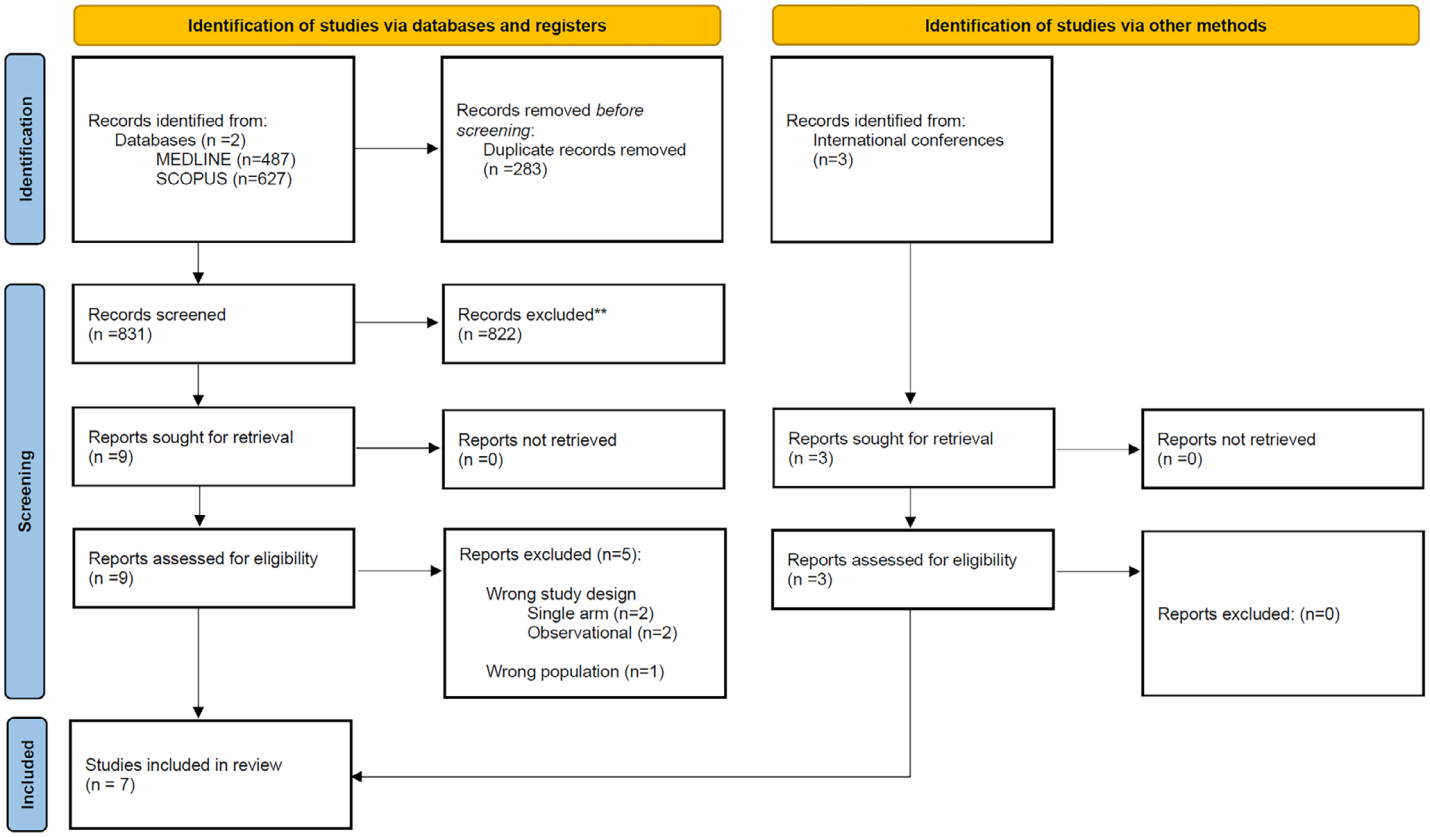
Flow chart of the systematic review.

The systematic database search yielded a total of 487 and 627 records from the MEDLINE and SCOPUS databases, respectively (Figure 1). After excluding duplicates and initial screening, we retrieved the full‐text of 9 records that were considered potentially eligible for inclusion. After reading the full‐text articles, 5 were further excluded (Table S1). Furthermore, after searching the reference lists of published articles and international conference abstracts, 3 additional records were included. Finally, we identified 7 eligible RCTs for inclusion in the systematic review and meta‐analysis (Table 1) [23, 24, 25, 26, 27, 28, 29, 30, 31], comprising a total of 2131 AIS patients with LVO and successful recanalization following EVT, receiving either IAT together with BMT [*n* = 1083; mean age 68.1 years; 36% female; mean baseline National Institutes of Health Stroke Scale (NIHSS) score of 16] versus BMT alone (*n* = 1048; mean age 68.4 years; 41% female, mean baseline NIHSS‐score of 16; Figures S1–S3). IVT before EVT was permitted in two RCTs, among which 52% received IVT in the intervention group versus 50% in the control group (Figure S4). There were no significant baseline differences in the two groups. Of the seven included RCTs, six focused exclusively on patients with anterior circulation LVO [23, 24, 26, 27, 28, 29, 30, 31], while one trial enrolled only patients with posterior circulation occlusions [25]. Geographically, six of the seven trials were conducted in China [23, 24, 25, 27, 28, 29, 30, 31], and one trial was conducted in Spain [26]. Regarding the thrombolytic agents used, four RCTs investigated intra‐arterial tenecteplase [23, 24, 25, 27, 30], two used intra‐arterial alteplase [26, 28, 29], and one evaluated intra‐arterial urokinase [31].

**TABLE 1:** Baseline characteristics of studies included in the systematic review and meta‐analysis.

Study	Country	Recruitment period	Thrombolytic drug; dose (% licensed full‐dose for IVT)	Inclusion criteria	Group	Age (years)	Female (%)	NIHSS	Vessel occlusion (n)				eTICI (n)			IVT (%)
									ICA	M1	M2	PCA	2b	2c	3	
ANGEL‐TNK [23, 24] (Phase 4)	China	Jan 2023–Mar 2024	Tenecteplase 0.125 mg/kg (50%)	pre‐mRS < 2; NIHSS > 1; eTICI: 2b50‐3; Within 4.5–24 h from onset; Ischemic core on CT perfusion < 70 mL	IAT (*n* = 126)	71.5 (60.6–79.5)	46	15 (12–19)	33	58	35	—	87	39		—
					BMT (*n* = 129)	71.7 (61.7–79)	43	16 (12–19)	38	64	27	—	77	50		—
ATTENTION‐IA [25] (Phase 2–3)	China	Jan 2023–Aug 2023	Tenecteplase 0.0625 mg/kg (25%)	pre‐mRS < 2; NIHSS > 5; eTICI: 2b50 to 3; Within 24 h from onset; pc‐ASPECTS > 5; occlusions in the basilar artery, vertebral artery or P1	IAT (*n* = 104)	65 ± 11.3	19	20 (12–35)	—	—	—	104	20	12	72	—
					BMT (*n* = 104)	67.3 ± 10.8	30	23 (14–35)	—	—	—	104	10	16	78	—
CHOICE [26] (Phase 2b)	Spain	Dec 2018–May 2021	Alteplase 0.0225 mg/kg (25%)	pre‐mRS < 2; NIHSS< 26; eTICI: 2b50 to 3; Within 24 h from symptoms onset; ASPECTS > 5;	IAT (*n* = 61)	73 (71–76)	46	14 (8–20)	13	19	33	—	34	27		62
					PCB (*n* = 52)	73 (69–77)	46	14 (10–20)	8	20	28	—	31	21		60
DATE [27] (Phase 2a)	China	Feb 2024–Dec 2024	Tenecteplase 0.03125 mg/kg (12.5%) Or 0.0625 mg/kg (25%)	pre‐mRS < 2; NIHSS 5–24; eTICI 2b50‐3; Within 24 h from symptoms onset; ASPECTS > 5	IAT (*n* = 92)	71	36	17 (12–20)	13	66	13	—	23	33	36	—
					BMT (*n* = 65)	71 (56–78)	54	17 (12–20)	25	35	5	—	17	21	27	—
PEARL [28, 29] (Phase 3)	China	not reported	Alteplase 0.225 mg/kg (25%)	pre‐mRS: < 2; NIHSS 6–25; eTICI 2b50‐3; Within 24 h from symptoms onset; ASPECTS > 5	IAT (*n* = 164)	65.1 ± 12.9	27	15 (11–17)	25	113	26	—	99	56	9	42
					BMT (*n* = 160)	66.5 ± 12.6	34	15 (11–18)	20	121	19	—	99	51	20	41
POST‐TNK [30] (Phase 3)	China	Oct 2022‐ Mar 2024	Tenecteplase 0.0625 mg/kg (25%)	pre‐mRS < 2; NIHSS < 26; eTICI: 2c to 3; Within 24 h from symptoms onset; ASPECTS > 5	IAT (*n* = 269)	69 (59–76)	43	15 (11–20)	57	172	40	—	—	101	167	—
					BMT (*n* = 271)	69 (59–76)	39	15 (10–20)	59	166	46	—	—	102	167	—
POST‐UK [31] (Phase 3)	China	Nov 2022–Mar 2024	Urokinase 100.000 IU (7%–10%)	pre‐mRS < 2; NIHSS < 26; eTICI 2c to 3; Within 24 h from symptoms onset; ASPECTS > 5	IAT (*n* = 267)	69 (59–77)	39	15 (11–19)	57	161	49	—	—	96	168	—
					BMT (*n* = 267)	68 (58–76)	44	15 (10–19)	68	148	51	—	—	90	175	—

Abbreviations: ASPECTS, Alberta Stroke Program Early CT Score; BMT, best medical treatment; eTICI, expanded treatment in cerebral infarction; IAT, intra‐arterial thrombolysis; ICA, internal carotid artery; IVT, intravenous thrombolysis; M1, M1 segment of the middle cerebral artery; M2, M2 segment of the middle cerebral artery; mRS, modified Rankin Scale; NIHSS, National Institutes of Health Stroke Scale; P1, P1 segment of the posterior cerebral artery; PCA, posterior circulation artery; pc‐ASPECTS, posterior circulation Alberta Stroke Program Early CT Score.

### 3.2 Quality Control of Included Studies

The assessment for the risk of bias in the included RCTs is presented in Figure S5. All studies utilized an appropriate randomization process resulting in low risk of bias in that domain. The CHOICE trial used placebo in the control group [26] and the ATTENTION‐IA trial did not present deviations from the intended intervention [25], while the rest of the trials had some concerns in that domain, attributed to minor deviations from intended intervention in an unblinded setting. The DATE [27], PEARL [28, 29], POST‐TNK [30], and POST‐UK [31] had some concerns in the third domain because of missing outcome data; however, they did not exceed 10% of the enrolled population. All studies presented low risk of bias in the measurement of the outcomes, as the investigators were blinded to the treatment each patient had received. The risk of bias due to reporting of outcomes was low in all included studies.

### 3.3 Quantitative Analyses

An overview of analyses for primary and secondary outcomes is summarized in Table 2.

**TABLE 2:** Overview of analyses for the outcomes of interest.

Outcome	Effect measure	Value (95% CI)	*p*	Prediction interval	*N* of studies	*I* ^2^ (*p* for cochrane *Q*)	Subgroup differences
Primary efficacy outcome							
Excellent functional outcome	RR	1.23 (1.11–1.36)	< 0.001	1.08–1.40	7	0% (0.43)	0.20
Secondary efficacy outcomes							
Good functional outcome	RR	1.06 (0.98–1.15)	0.143	0.96–1.17	7	70% (0.51)	0.82
Reduced disability	cOR	1.10 (1.03–1.18)	0.005	1.01–1.20	7	0% (0.73)	0.22
Primary safety outcome							
Symptomatic intracranial hemorrhage	RR	1.14 (0.76–1.70)	0.525	0.67–1.93	6	0% (0.52)	0.48
Secondary safety outcome							
Any intracranial hemorrhage	RR	1.16 (0.98–1.37)	0.084	0.84–1.60	6	21% (0.28)	0.75
All‐cause mortality	RR	1.00 (0.83–1.21)	0.994	0.78–1.28	6	0% (0.45)	0.83

Abbreviations: CI, confidence interval; cOR, common odds ratio; RR, risk ratio.

**FIGURE 2:**
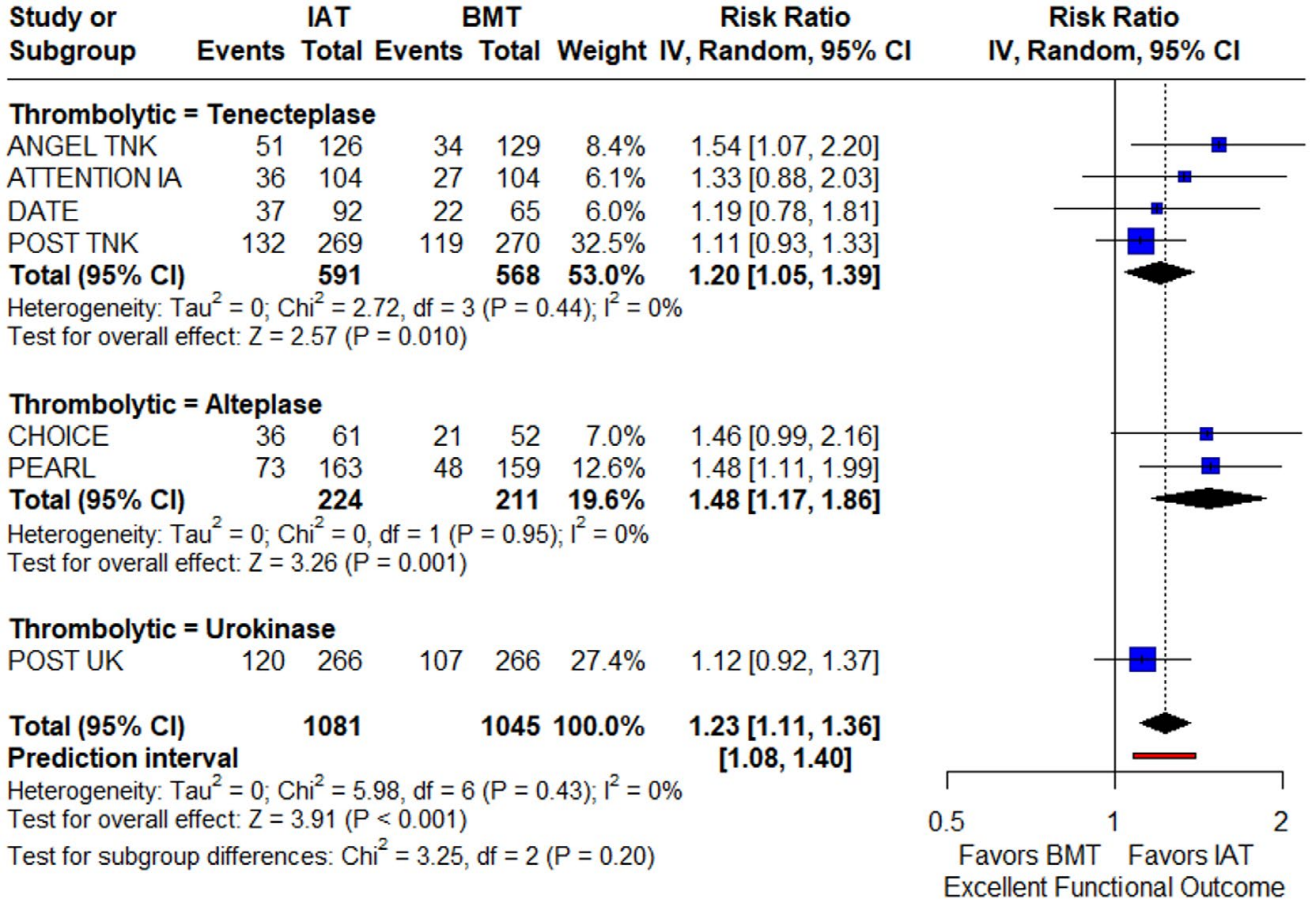
Forest plot presenting the risk ratio of excellent functional outcome at 3 months among patients receiving intra‐arterial thrombolysis (IAT) versus best medical treatment alone (BMT), stratified by the thrombolytic agent used.

**FIGURE 3:**
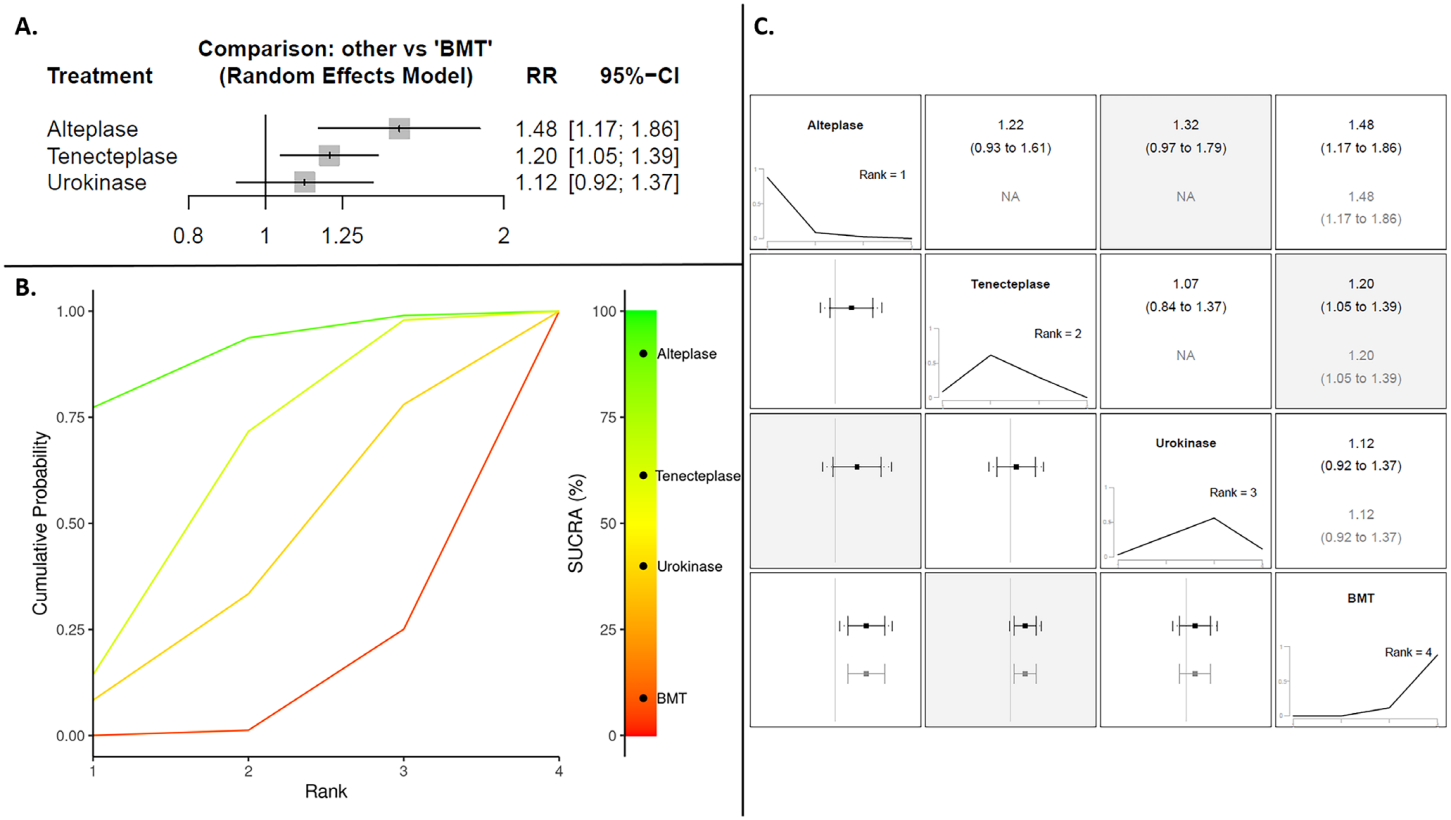
Forest plot of the random‐effects network meta‐analysis (NMA) presenting the risk ratio of excellent functional outcome at 3 months according to the treatment group, using best medical treatment (BMT) as reference (Panel A). SUCRA plot for the excellent functional outcome at 3 months with higher Surface Under the Cumulative Ranking Curve (SUCRA) values and cumulative ranking curves nearer the top left indicating better performance (Panel B). Summary forest plot for the risk ratio of excellent functional outcome at 3 months: NMA results in black; pairwise meta‐analysis results in gray; 95% confidence interval presented as error bars; interventions are ranked and sorted by SUCRA value.

Regarding the primary efficacy outcome, patients receiving IAT had significantly higher rates of achieving an excellent functional outcome at 3 months compared to those receiving BMT only (RR: 1.23; 95% CI: 1.11–1.36; *p* < 0.001; PI: 1.08–1.40; 7 studies; *I* ^2^ = 0%; *p* for Cochran *Q* = 0.43; Figure 2). The NNT for IAT was 12 (95% CI: 8–25). There were no significant subgroup differences between the different thrombolytics used (*p* for subgroup differences = 0.20). However, according to the NMA, IAT treatment with alteplase (RR: 1.48; 95% CI: 1.17–1.86) and tenecteplase (RR: 1.20; 95% CI: 1.05–1.39) were significantly associated with higher rates of excellent functional outcome compared to BMT alone, but urokinase was not (RR: 1.12; 95% CI: 0.92–1.37; Figure 3A). Alteplase had the highest SUCRA value (90%; Figure 3B) and ranked first among the different thrombolytics (Figure 3C), followed by tenecteplase (SUCRA value = 61%) and then by urokinase (SUCRA value = 40%). No evidence of inconsistency was observed during the NMA (*p* > 0.05). During sensitivity analysis stratifying by country of recruitment in each trial, there were no subgroup differences regarding the 3‐month excellent functional outcome (*p* for subgroup differences = 0.38; Figure S6). Finally, there was no treatment effect modification with IVT pretreatment (*p* for subgroup differences = 0.62; Figure S7A), relevant dose of thrombolytic drug (*p* for subgroup differences = 0.46; Figure S7B), or degree of reperfusion (eTICI 2b vs. 2c/3; *p* for subgroup differences = 0.25; Figure S7C) for the primary efficacy outcome.

**FIGURE 4:**
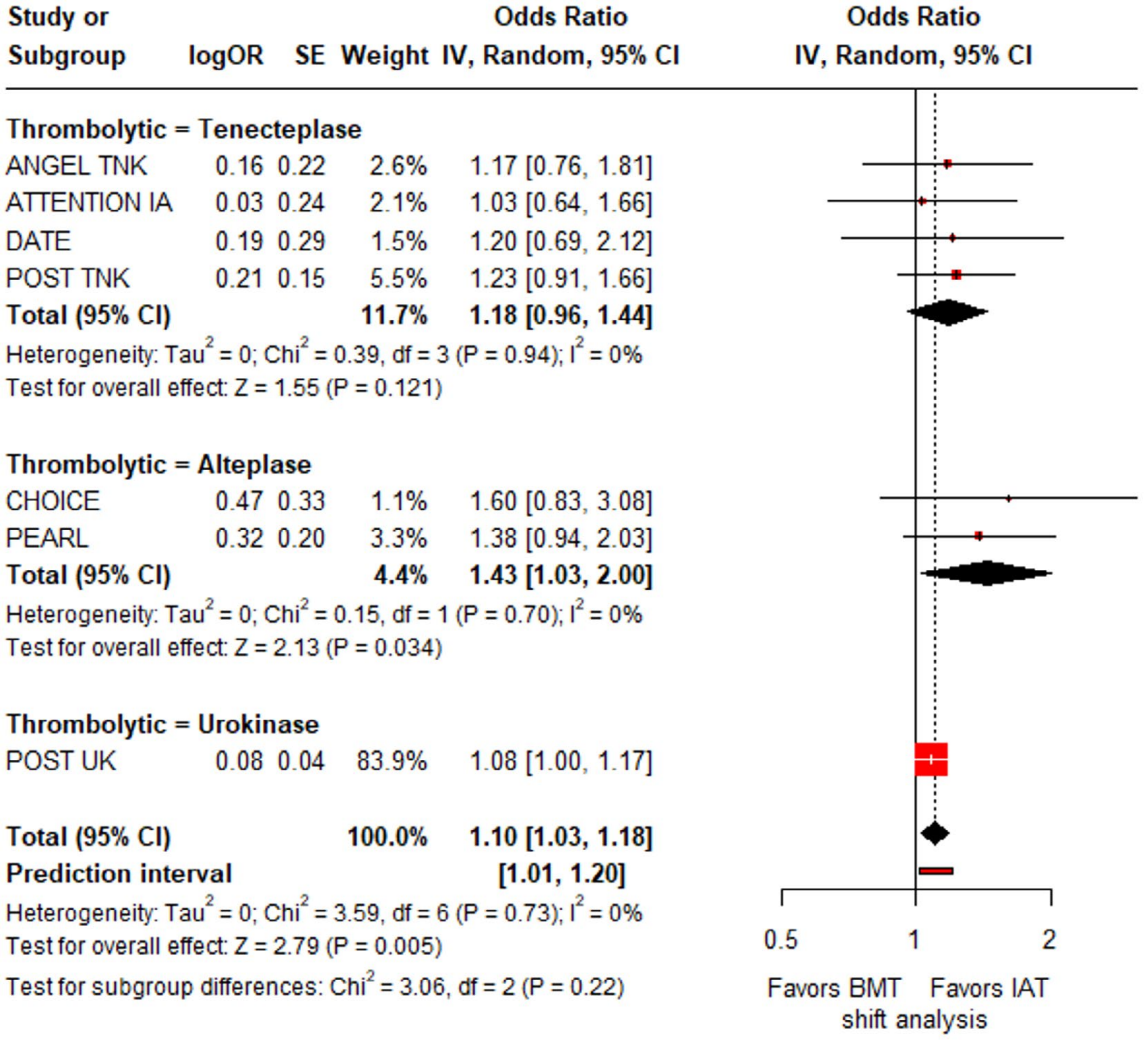
Forest plot presenting the common odds ratio of reduced disability at 3 months among patients receiving intra‐arterial thrombolysis (IAT) versus best medical treatment alone (BMT), stratified by the thrombolytic agent used.

Concerning secondary efficacy outcomes, similar rates of good functional outcome were noted for those receiving IAT with BMT compared to those receiving BMT alone (RR: 1.06; 95% CI: 0.98–1.15; *p* = 0.143; PI: 0.96–1.17; 7 studies; *I* ^2^ = 0%; *p* for Cochran *Q* = 0.51; Figure S8). However, IAT was associated with higher odds of reduced disability at 3 months compared to BMT alone (cOR: 1.10; 95% CI: 1.03–1.18; *p* = 0.005; PI: 1.01–1.20; 7 studies; *I* ^2^ = 0%; *p* for Cochran *Q* = 0.73; Figure 4). There were no differences among the different thrombolytic agents in any of those outcomes.

**FIGURE 5:**
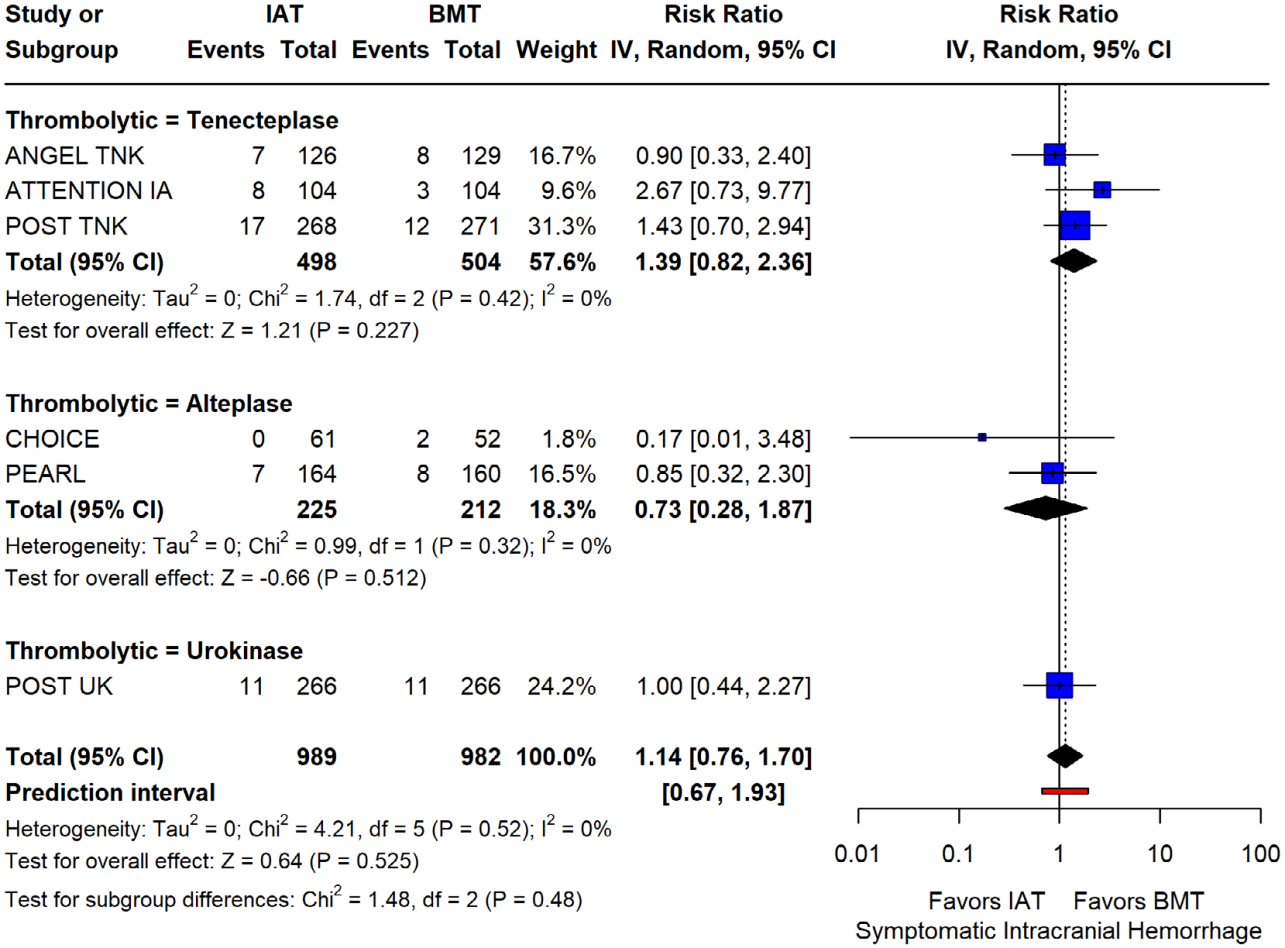
Forest plot presenting the risk ratio of symptomatic intracranial hemorrhage among patients receiving intra‐arterial thrombolysis (IAT) versus best medical treatment alone (BMT), stratified by the thrombolytic agent used.

Regarding safety outcomes, sICH was similar between IAT and BMT groups (RR: 1.14; 95% CI: 0.76–1.70; *p* = 0.988; PI: 0.67–1.93; 6 studies; *I* ^2^ = 0%; *p* for Cochran *Q* = 0.52; Figure 5). There were no significant subgroup differences between the different thrombolytics used (*p* for subgroup differences = 0.48). Similarly, there was no difference for any‐ICH between the IAT and the BMT groups (RR: 1.16; 95% CI: 0.98–1.37; *p* = 0.084; PI: 0.84–1.60; 6 studies; *I* ^2^ = 21%; *p* for Cochran *Q* = 0.28; Figure S9). Finally, all‐cause mortality at 3 months was similar between the two groups (RR: 1.00; 95% CI: 0.83–1.21; *p* = 0.994; PI: 0.78–1.28; 6 studies; *I* ^2^ = 0%; *p* for Cochran *Q* = 0.45; Figure S10).

The pooled proportions per arm for each outcome of interest are presented in Table S2.

In the assessment of publication bias, no asymmetry was noted for any of the outcomes during inspection of the funnel plots (Figures S11–S16).

## 4 Discussion

In this systematic review and meta‐analysis of seven RCTs comprising 2131 AIS patients with anterior or posterior circulation LVO and successful recanalization following EVT, we found that adjunct IAT was associated with a significantly higher likelihood of achieving excellent functional outcomes and higher odds of reduced disability at 90 days, without a concomitant increase in adverse safety events, including sICH, any‐ICH, and 3‐month all‐cause mortality. These findings support the potential role of IAT as a targeted post‐recanalization strategy to improve neurological recovery in patients with AIS due to LVO.

Our primary efficacy analysis demonstrated that IAT significantly increased the probability of excellent functional outcome compared to BMT alone, with a RR of 1.23 and a NNT of 12. These findings are clinically meaningful, particularly in the context of the persistent therapeutic gap seen even among patients who achieve successful large‐vessel recanalization. Notably, the proportion of excellent outcomes in the control (BMT only after EVT) group was 34%, aligning with historical data that underscore the high rate of functional dependency despite angiographic success [5].

Moreover, while IAT did not significantly improve rates of good functional outcome, it was associated with a modest but statistically significant increase in disability reduction, as evidenced by the favorable shift across the entire mRS spectrum. This observation reflects a nuanced benefit of IAT that may be more pronounced in promoting meaningful neurological recovery rather than merely crossing arbitrary functional thresholds.

Safety findings were equally reassuring. The rates of sICH, any‐ICH, and all‐cause mortality at 90 days were comparable between IAT and BMT groups. More specifically, the pooled proportion of sICH in the IAT group was 4%, which is comparable to the one reported in the HERMES collaboration including patients with EVT without adjunctive IAT [5]. These results reinforce the notion that when administered even in a postthrombectomy context, IAT does not appear to increase bleeding complications or mortality risk [32]. This safety profile is critical for the broader acceptance and potential implementation of IAT in standard stroke workflows.

Our network meta‐analysis provides additional insights into the relative efficacy of different intra‐arterial thrombolytic agents. Specifically, both intra‐arterial alteplase and tenecteplase were associated with improved functional outcomes, whereas urokinase demonstrated a more neutral effect. Among the agents, alteplase ranked highest in terms of efficacy, as reflected by its superior SUCRA value. However, these findings should be interpreted with caution, as only one RCT evaluated urokinase, and substantial variability existed across studies in terms of dosing regimens, pharmacokinetics, and patient populations. Furthermore, only two RCTs [26, 28]—both utilizing intra‐arterial alteplase—permitted IVT with alteplase prior to EVT in a subset of participants. This may have influenced the observed effect of IAT and introduces an additional layer of heterogeneity in baseline treatment exposure. Yet, our subgroup analysis found no evidence that IVT pretreatment modified the effect of adjunctive IAT on excellent functional outcome.

In addition, further subgroup analyses were conducted to evaluate whether the efficacy of adjunctive IAT varied by the degree of macrovascular reperfusion (eTICI 2b vs. 2c/3) or by thrombolytic dose (% of full‐dose approved for IVT). Neither analysis demonstrated a statistically significant treatment effect modification on excellent functional outcome. These findings imply that the benefit of IAT may extend across a spectrum of reperfusion grades and support the hypothesis that enhancing microvascular perfusion could contribute to improved outcomes, even in patients with near‐complete angiographic reperfusion. Importantly, the absence of a clear dose–response signal highlights the need for further trials to define the optimal dosing strategy for IAT.

Despite low statistical heterogeneity across most outcomes and although subgroup analyses stratified by thrombolytic agent or dose, reperfusion status, or administration of IVT revealed no significant treatment effect modifications, further clinical and procedural differences were present across the included RCTs. Inclusion criteria varied in terms of baseline NIHSS thresholds, timing of IAT administration, and baseline imaging protocols. These differences may have influenced treatment responsiveness and could limit the generalizability of our pooled estimates to certain patient subgroups or healthcare settings. Future trials should adopt more standardized eligibility and procedural criteria to allow for more homogeneous comparisons and improve external validity.

Several ongoing RCTs are expected to further clarify the utility of IAT in stroke care, such as the Intra‐Arterial Thrombolysis After SUCCESSful Reperfusion in Anterior Circulation Ischemic Stroke (IA‐SUCCESS; NCT06768138), the Intra‐arterial Thrombolysis After Successful Thrombectomy for Acute Ischemic Stroke in the Posterior Circulation (IAT‐TOP; NCT05897554), and the CHemical OptImization of Cerebral Embolectomy 2 (CHOICE 2; NCT05797792) trials. As these data mature, they may help refine the role of adjunct thrombolysis, particularly in underexplored populations such as patients with occlusions of the posterior circulation and those with incomplete reperfusion or microvascular deficits on perfusion imaging, where randomized evidence remains scarce. Upcoming secondary analyses from included trials may also inform critical procedural variables including thrombolytic selection, dosing strategies, and timing of administration.

In parallel, there is growing interest in expanding the use of IAT to patients without successful recanalization—an area that remains investigational but conceptually compelling given the pathophysiological relevance of distal emboli and microvascular no‐reflow [33]. Recently, a single‐arm pilot trial has shown that adjunctive intra‐arterial tenecteplase in AIS patients with incomplete reperfusion (modified TICI 2b‐2c) post‐EVT was feasible and was not associated with increased rates of intracranial hemorrhage [34]. Several RCTs in this setting, including the Safety and Efficacy of Intra‐arterial Tenecteplase for Noncomplete Reperfusion of Intracranial Occlusions (TECNO; NCT05499832), the Improving Neuroprotective Strategy for Ischemic Stroke With Poor Recanalization After Thrombectomy by Intra‐arterial TNK (INSIST‐TNK; NCT04201964), and the Intra‐arterial Tenecteplase During First Thrombectomy Attempt for Acute Stroke (BRETIS‐TNK II; NCT05657444) [35] trials are ongoing or with results awaited.

Our findings are largely consistent with those of prior meta‐analyses [36, 37]; however, several important distinctions warrant emphasis. First, our meta‐analysis includes all available RCTs with reported results to date, including those presented at major scientific meetings, ensuring a comprehensive and up‐to‐date synthesis of the evidence. Second, we performed both pairwise and network meta‐analyses, enabling a more nuanced assessment of agent‐specific effects across different thrombolytics. Third, the use of prediction intervals in our analyses adds an additional layer of methodological rigor by providing estimates of the expected range of treatment effects in future studies, thereby enhancing the clinical interpretability and reproducibility of our findings.

Notwithstanding its strengths, this meta‐analysis has limitations. Despite restricting inclusion to RCTs, the overall number of eligible studies remains modest (*n* = 7), and differences in inclusion criteria, imaging protocols, thrombolytic agents, and outcome definitions introduce potential heterogeneity. Additionally, for three of the included RCTs, data were extracted from oral presentations at the International Stroke Conference 2025 [23, 27, 29], as detailed study results were not yet published at the time of this analysis. Nevertheless, the primary outcome data are unlikely to change substantially in the final publications. Furthermore, although statistical heterogeneity across most endpoints was low (*I* ^2^ = 0% in most analyses), clinical and methodological variability remains a concern. Specifically, differences among the included RCTs in intra‐arterial thrombolytic agents and dosing regimens, eligibility for IVT, inclusion criteria thresholds (e.g., varying definitions of successful reperfusion and different treatment time windows), as well as the predominant recruitment of Chinese populations, may limit the broader generalizability and introduce potential confounding. Our reliance on aggregate‐level data precluded detailed subgroup analyses and adjustment for individual patient characteristics, such as collateral status, infarct core volume, and postprocedural perfusion metrics. Future individual patient data meta‐analyses will be critical to delineate nuanced treatment effects, particularly in relation to imaging biomarkers and demographic modifiers such as age, sex, or comorbidity profiles.

## 5 Conclusions

In conclusion, IAT appears to be a safe and effective adjunct to EVT in AIS patients with successful recanalization, conferring improved rates of excellent functional outcomes without increasing the risk of intracranial hemorrhage or death. As additional trial data emerges, IAT may become a valuable tool in optimizing microvascular reperfusion and functional outcomes after EVT.

## Author Contributions

**Lina Palaiodimou:** data curation, formal analysis, investigation, methodology, visualization, writing – original draft. **Nikolaos M. Papageorgiou:** methodology, investigation, formal analysis, writing – original draft, data curation, visualization. **Guillaume Turc:** data curation, writing – review and editing. **Benjamin Gory:** data curation, writing – review and editing. **Aikaterini Theodorou:** methodology, data curation, investigation, writing – review and editing. **Eleni Bakola:** data curation, methodology, investigation, writing – review and editing. **George Magoufis:** data curation, writing – review and editing. **Stavros Spiliopoulos:** data curation, writing – review and editing. **Michail Mantatzis:** data curation, writing – review and editing. **Nitin Goyal:** data curation, writing – review and editing. **Marios Themistocleous:** data curation, writing – review and editing. **Amrou Sarraj:** data curation, writing – review and editing. **Aristeidis H. Katsanos:** data curation, writing – review and editing. **Urs Fischer:** data curation, writing – review and editing. **Andrei V. Alexandrov:** data curation, writing – review and editing. **Georgios Tsivgoulis:** conceptualization, methodology, data curation, investigation, formal analysis, supervision, project administration, writing – original draft.

## Ethics Statement

The authors have nothing to report.

## Consent

The authors have nothing to report.

## Conflicts of Interest

The authors declare no conflicts of interest.

## Supporting information


Data S1.

## Data Availability

All data generated or analyzed during this study are included in this article and its supplementary files.
