# Supplementary material for: The Added Benefit of Intra‐Arterial Thrombolysis After Successful Recanalization by Endovascular Treatment: A Systematic Review and Meta‐Analysis of Randomized‐Controlled Clinical Trials
Source: Eur J Neurol. 2025 Jul 7;32(7):e70270. doi: 10.1111/ene.70270 (PMC12231048; doi:10.1111/ene.70270)

**Supplement:**

**eMethods**

**eTables 1-2**

**eReferences**

**eFigures 1-16**

**eMethods**

**Complete search algorithm used in MEDLINE (using PubMed).**

"ischemic stroke"[Title/Abstract] OR "AIS"[Title/Abstract]

AND ("thrombectomy"[Title/Abstract] OR "EVT"[Title/Abstract] OR "endovascular treatment"[Title/Abstract] OR "mechanical thrombectomy"[Title/Abstract])

AND ("intra-arterial thrombolysis"[Title/Abstract] OR "intraarterial thrombolysis"[Title/Abstract] OR "IA thrombolysis"[Title/Abstract] OR "adjunctive thrombolysis"[Title/Abstract] OR "thrombolysis"[Title/Abstract])

AND ("modified Rankin Scale"[Title/Abstract] OR "mRS"[Title/Abstract] OR "functional outcome"[Title/Abstract])

AND ("successful recanalization"[Title/Abstract] OR "TICI"[Title/Abstract])

**Complete search algorithm used in Scopus.**

(TITLE-ABS-KEY("ischemic stroke" OR "AIS"))

AND

(TITLE-ABS-KEY("thrombectomy" OR "EVT" OR "endovascular treatment" OR "mechanical thrombectomy"))

AND

(TITLE-ABS-KEY("intra-arterial thrombolysis" OR "intraarterial thrombolysis" OR "IA thrombolysis" OR "adjunctive thrombolysis" OR "thrombolysis"))

AND

(TITLE-ABS-KEY("modified Rankin Scale" OR "mRS" OR "functional outcome"))

AND

(TITLE-ABS-KEY("successful recanalization" OR "TICI"))

**eTable 1:** Excluded studies with reasons of exclusion

| **Study Name** | **Reason for Exclusion** |
| --- | --- |
| Collette et al. ^1^ | Wrong study design – Observational |
| Furlan et al. ^2^ | Wrong population |
| Qureshi et al. ^3^ | Wrong study design – Observational |
| Zaidi et al. ^4^ | Wrong study design – Single arm |
| Zhao et al. ^5^ | Wrong study design – Single arm |

**eTable 2:** Pooled proportions per arm for each outcome of interest.

| **Outcomes of interest** | **Pooled Proportion (95% Confidence Interval)** | |
| --- | --- | --- |
|  | **IAT** | **BMT** |
| **Primary Efficacy Outcome** | | |
| Excellent Functional Outcome | 45% (40-49%) | 34% (29-40%) |
| **Secondary Efficacy Outcome** | | |
| Good functional outcome | 54% (47-60%) | 49% (43-56%) |
| **Primary Safety Outcome** | | |
| Symptomatic Intracranial Hemorrhage | 4% (2-7%) | 4% (3-6%) |
| **Secondary Safety Outcomes** |  |  |
| Any Intracranial Hemorrhage | 29% (25-34%) | 25% (21-30%) |
| All-cause mortality | 18% (14-22%) | 18% (14-22%) |

IAT: intra-arterial thrombolysis; BMT: best medical treatment

**eReferences.**

1. Collette SL, Bokkers RPH, Mazuri A, et al. Intra-arterial thrombolytics during endovascular thrombectomy for acute ischaemic stroke in the MR CLEAN Registry. *Stroke Vasc Neurol*. 2023;8:17-25.

2. Furlan A, Higashida R, Wechsler L, et al. Intra-arterial prourokinase for acute ischemic stroke. The PROACT II study: a randomized controlled trial. Prolyse in Acute Cerebral Thromboembolism. *Jama*. 1999;282:2003-2011.

3. Qureshi AI, Lodhi A, Ma X, et al. Intraarterial thrombolytics as an adjunct to mechanical thrombectomy in patients with basilar artery occlusion. *J Neuroimaging*. 2023;33:415-421.

4. Zaidi SF, Castonguay AC, Zaidat OO, et al. Safety of Adjunctive Intraarterial Tenecteplase Following Mechanical Thrombectomy: The ALLY Pilot Trial. *Stroke*. 2025;56:355-361.

5. Zhao ZA, Qiu J, Wang L, et al. Intra-arterial tenecteplase is safe and may improve the first-pass recanalization for acute ischemic stroke with large-artery atherosclerosis: the BRETIS-TNK trial. *Front Neurol*. 2023;14:1155269.

**eFigure 1.** Forest plots presenting the mean age (in years) among patients treated with intra-arterial thrombolysis (ΙΑΤ; Panel A), the mean age (in years) among patients treated with best medical treatment (BMT; Panel B), and the standardized mean difference of age (in years) among the patients treated with IAT versus BMT (Panel C).

**A.**


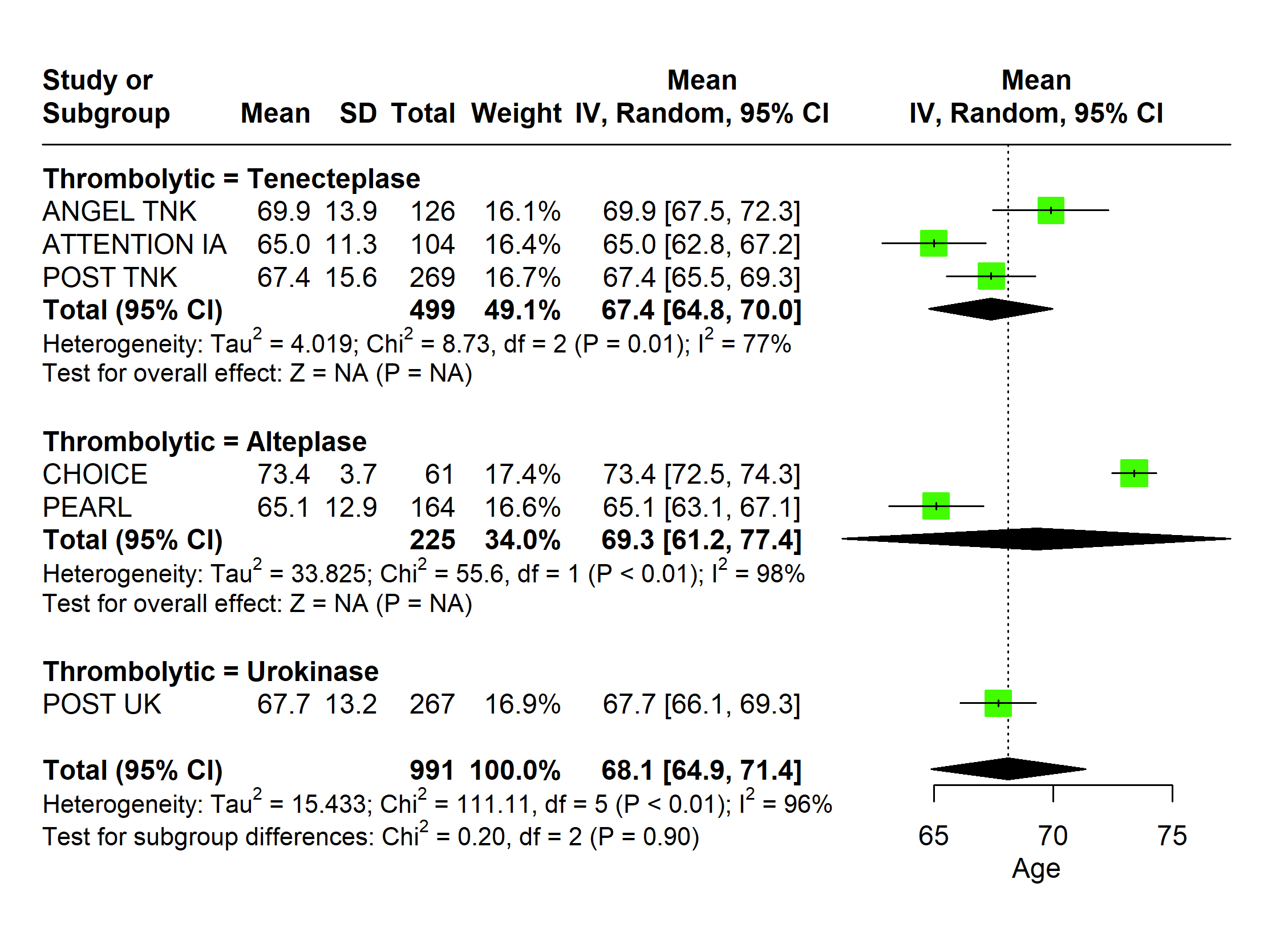


**B.**

**
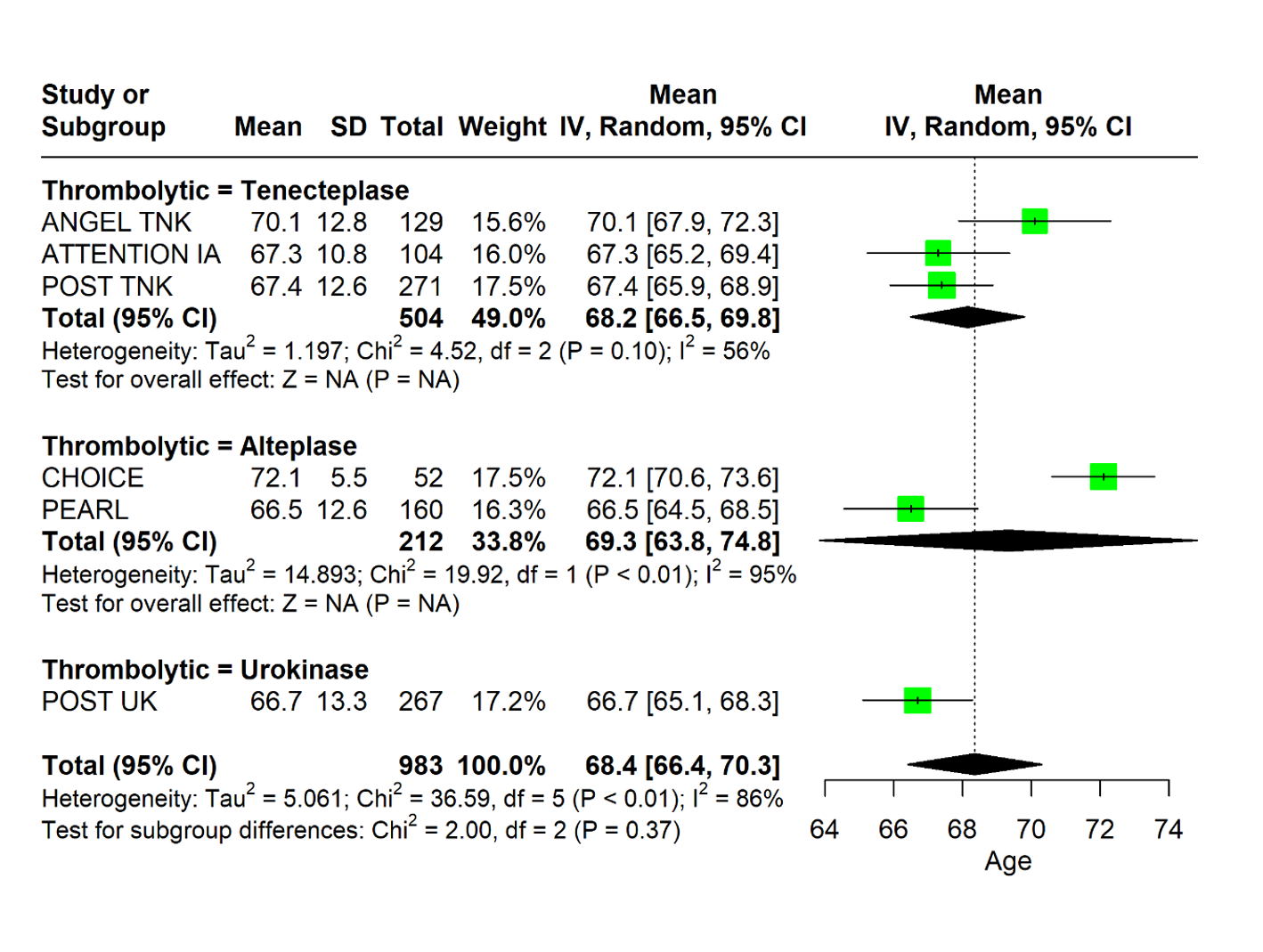
C.**

**
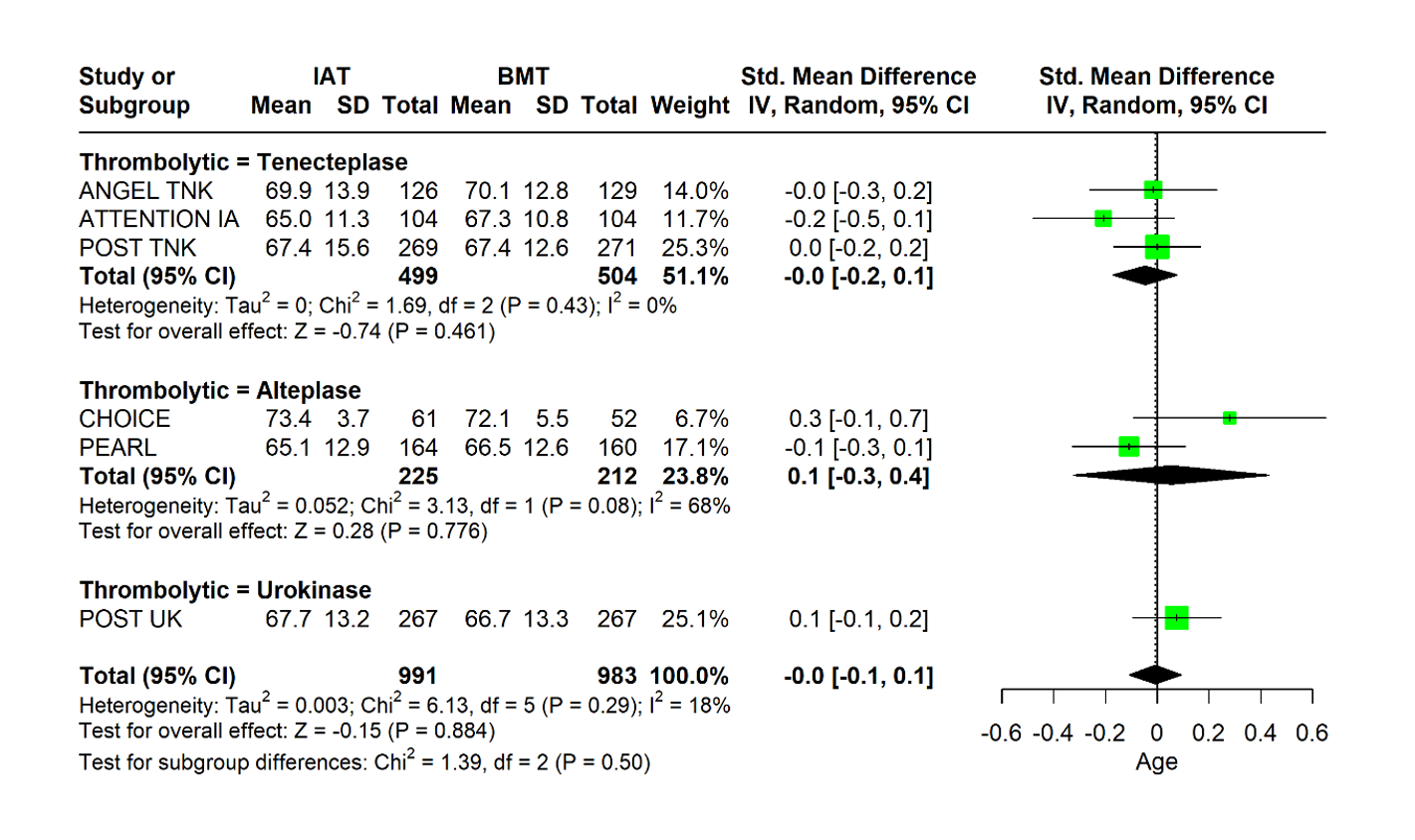
**

**eFigure 2.** Forest plots presenting the pooled proportion of female patients among those treated with intra-arterial thrombolysis (IAT; Panel A), the pooled proportion of female patients treated with best medical treatment (BMT; Panel B), and the odds ratio of female patients treated with IAT versus BMT (Panel C).

**A.**

**
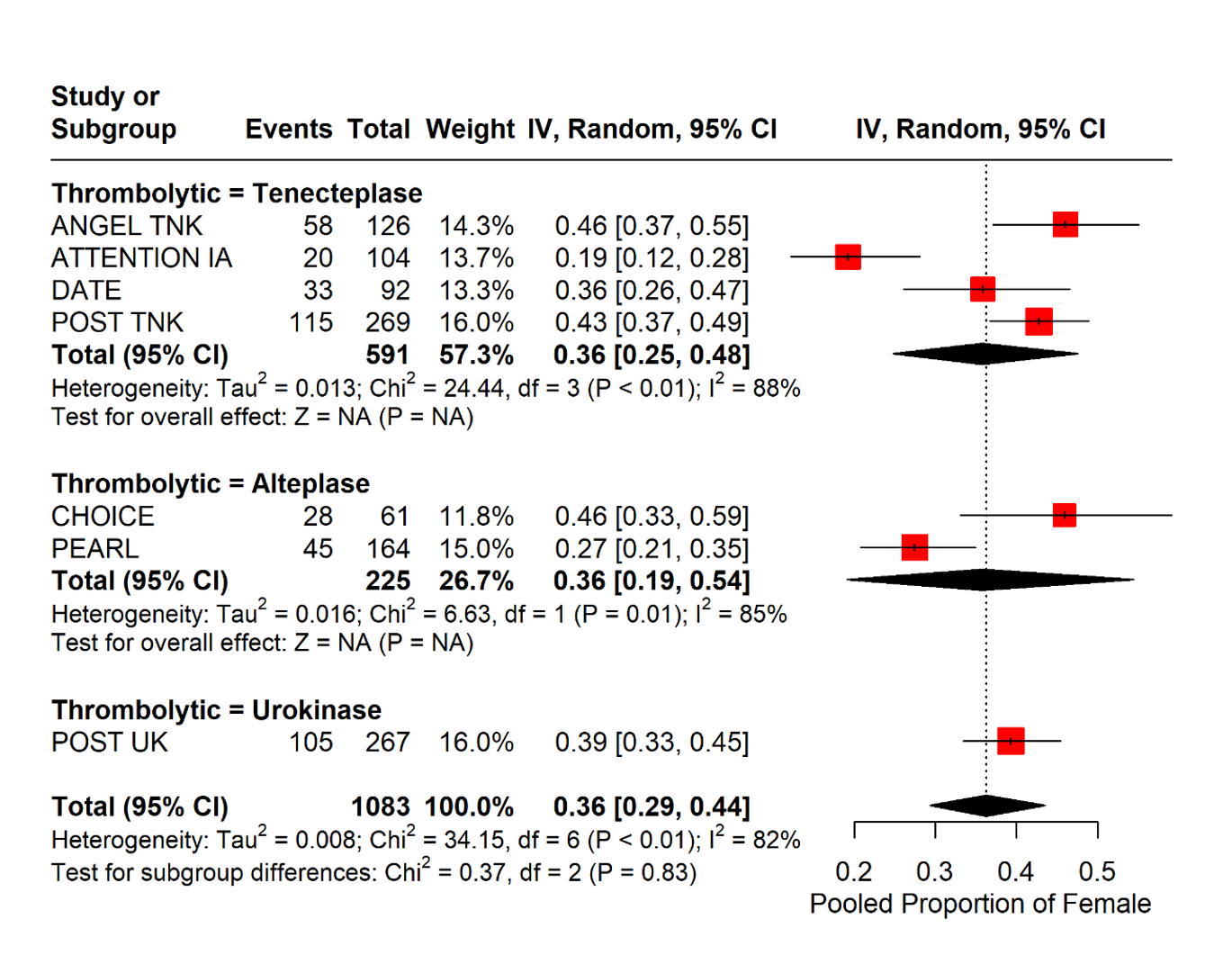
**

**B.**

**
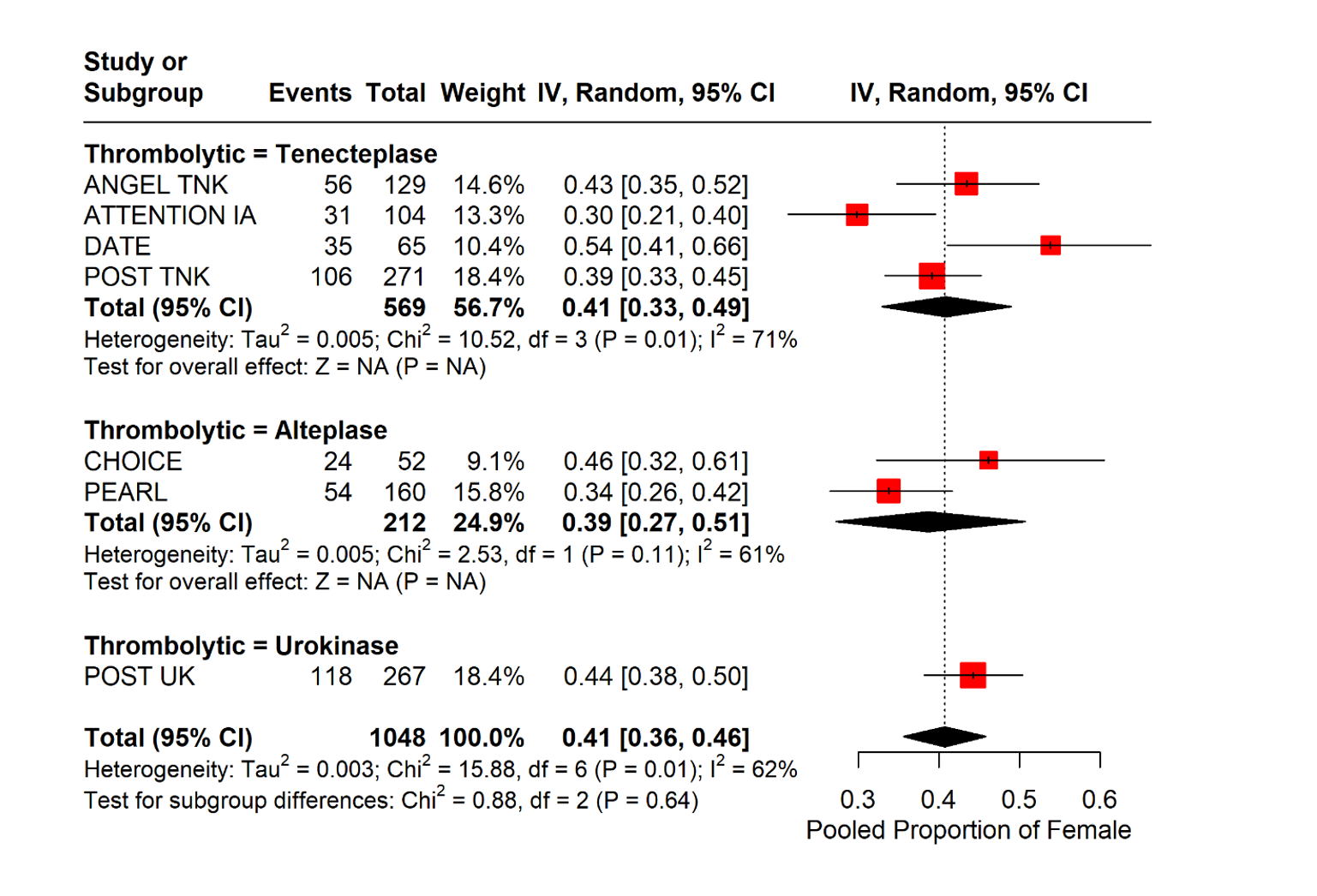
**

**C.**


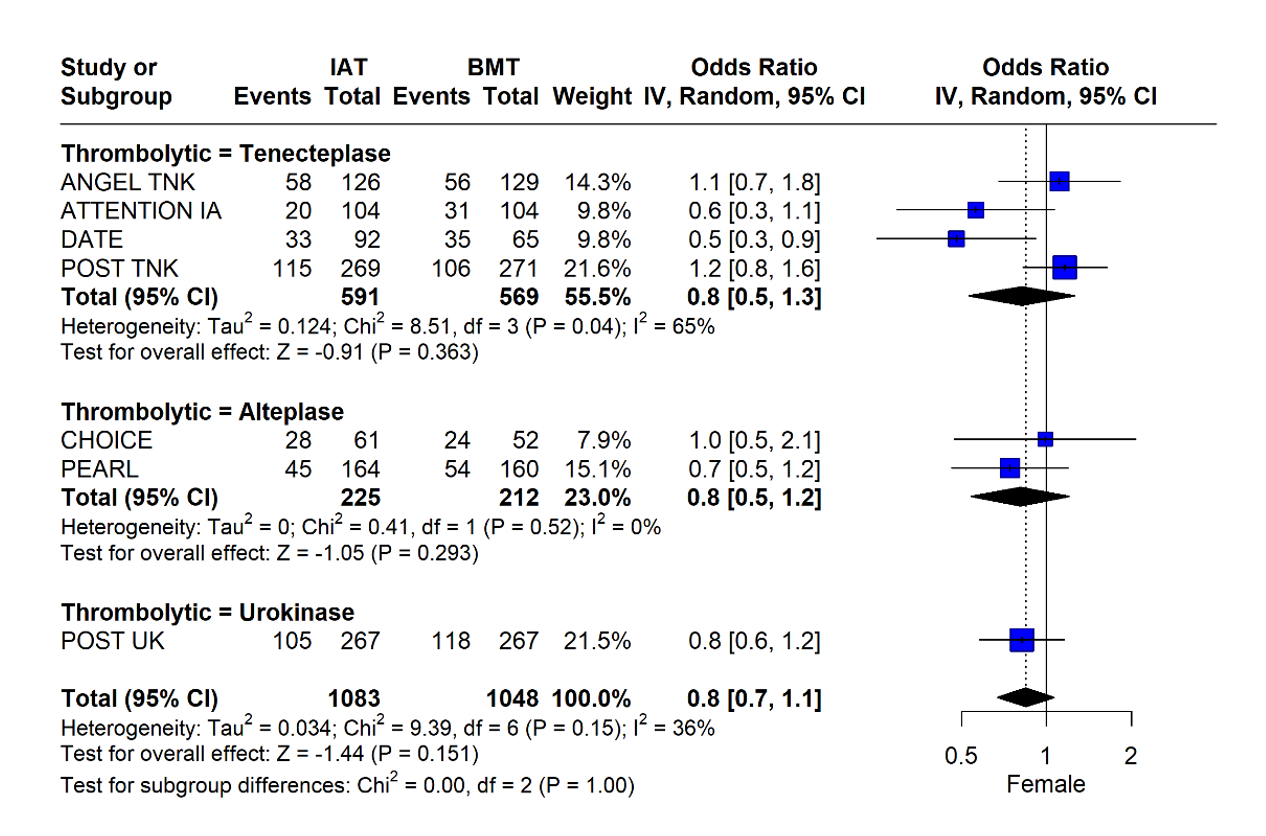


**eFigure 3.** Forest plots presenting the mean NIHSS score among patients treated with intra-arterial thrombolysis (ΙΑΤ; Panel A), the mean NIHSS score among patients treated with best medical treatment (BMT; Panel B), and the standardized mean difference of NIHSS score among the patients treated with IAT versus BMT (Panel C).

**A.**

**
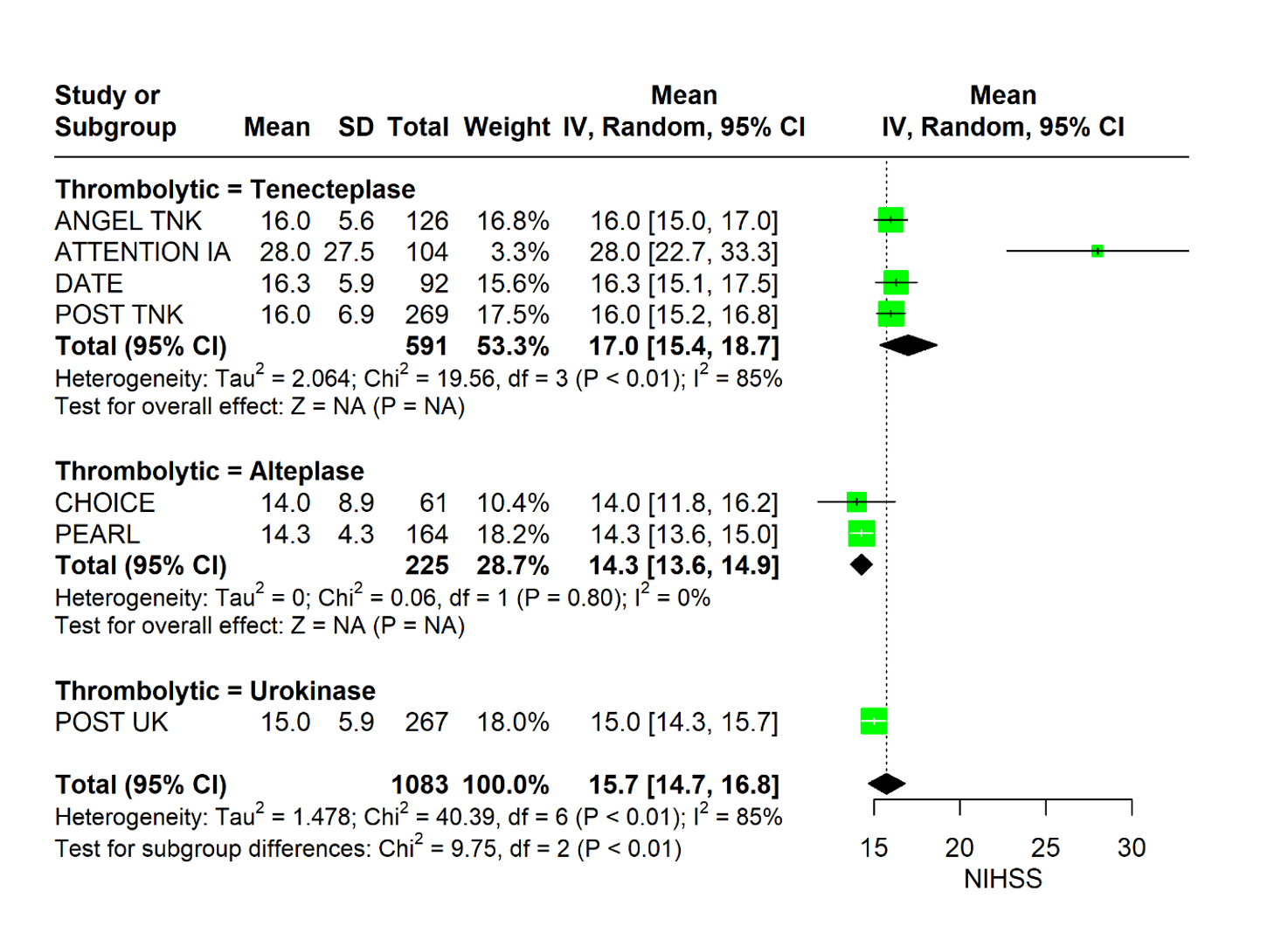
**

**B.**

**
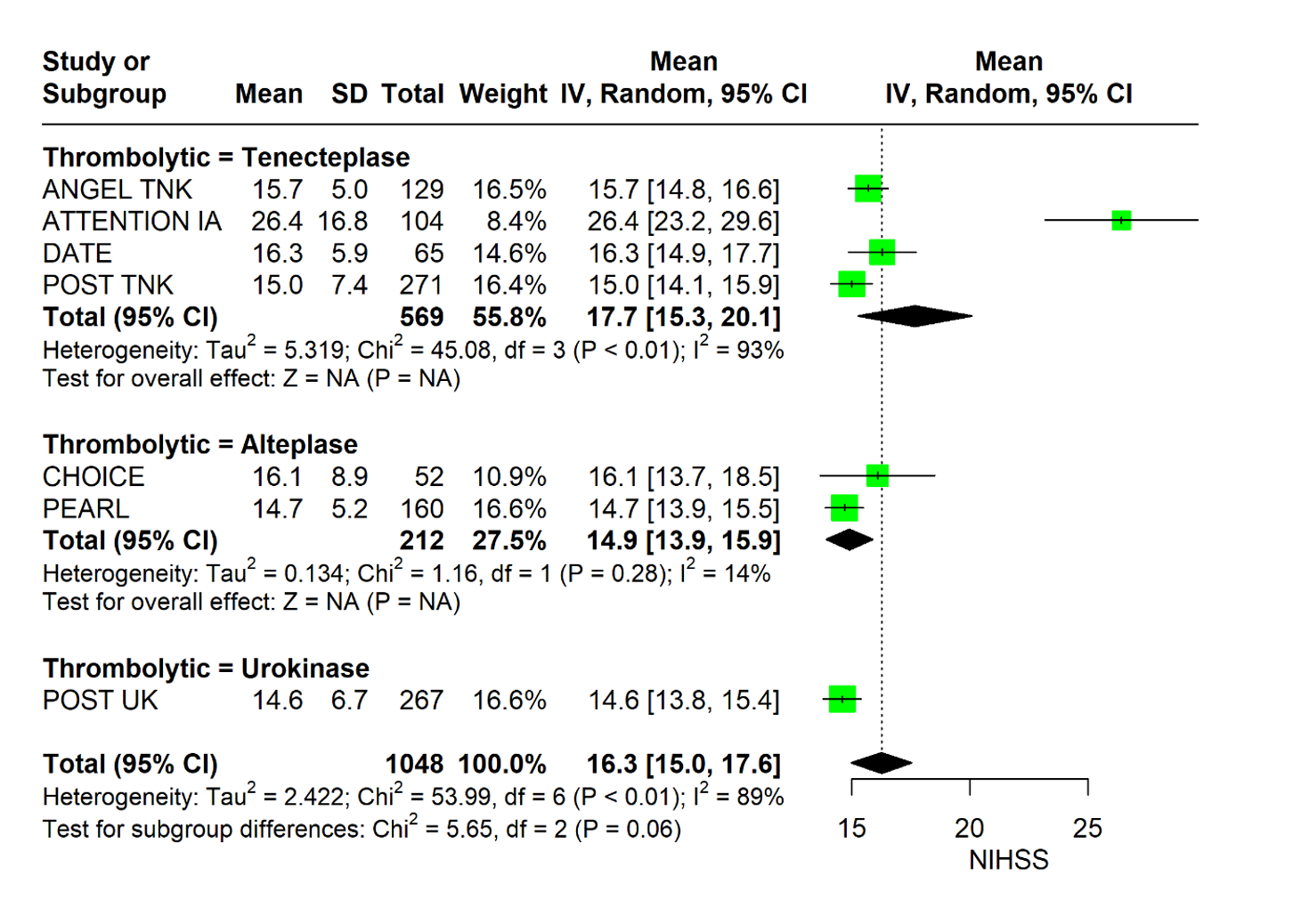
**

**C.**


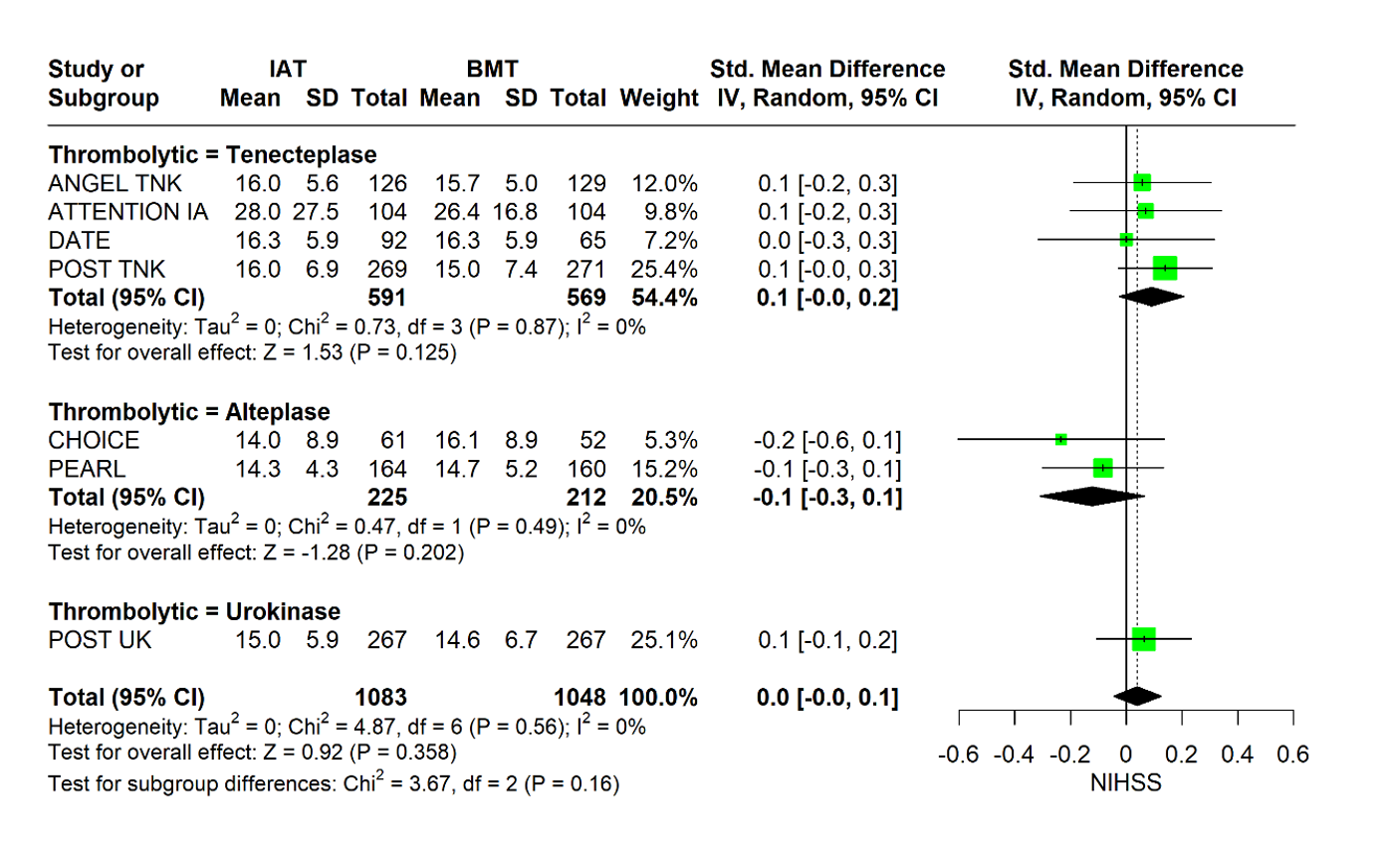


**eFigure 4.** Forest plots presenting the pooled proportion of patients receiving intravenous thrombolysis (IVT) among those treated with intra-arterial thrombolysis (IAT; Panel A), the pooled proportion of patients receiving IVT among those treated with best medical treatment (BMT; Panel B), and the odds ratio of IVT among patients treated with IAT versus BMT (Panel C).

**A.**


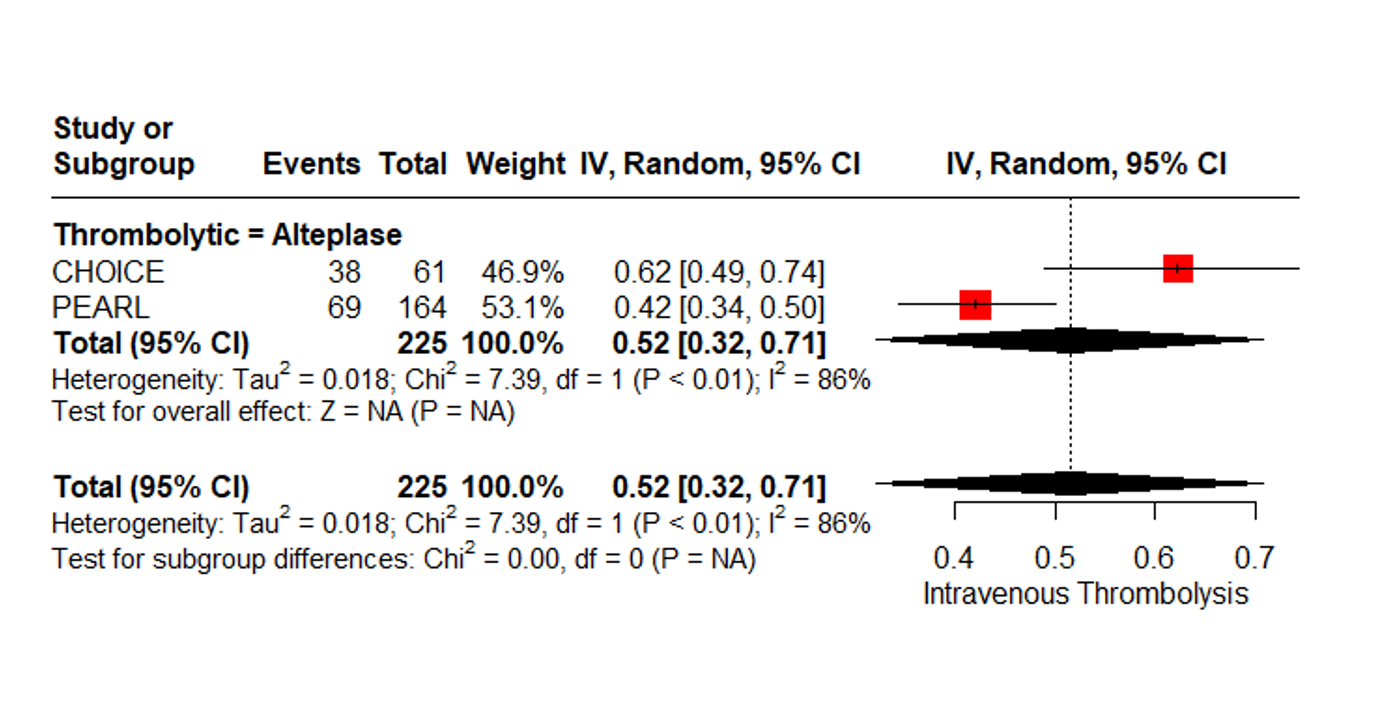


**B.**


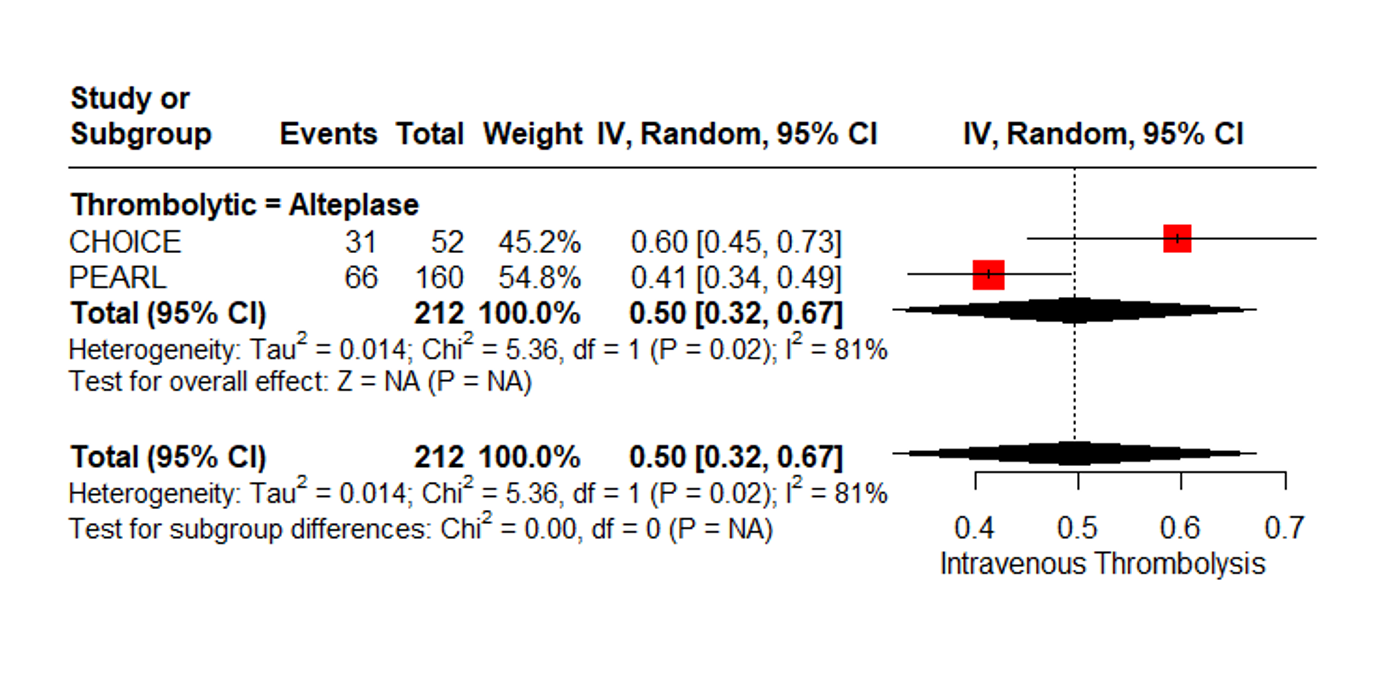


**C.**


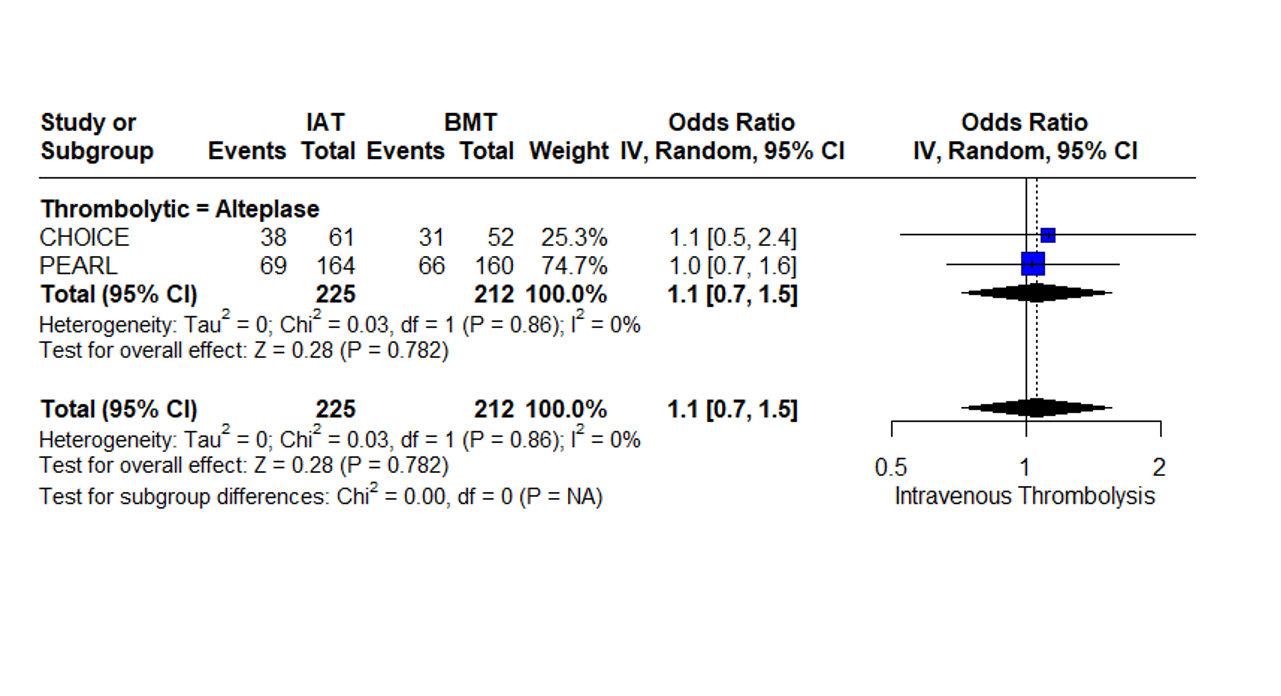


**eFigure 5.** Traffic light plot (A) and summary plot (B) presenting the quality assessment of the included randomized-controlled clinical trials (RCTs), using the Cochrane Collaboration tool (RoB 2).

**A.**


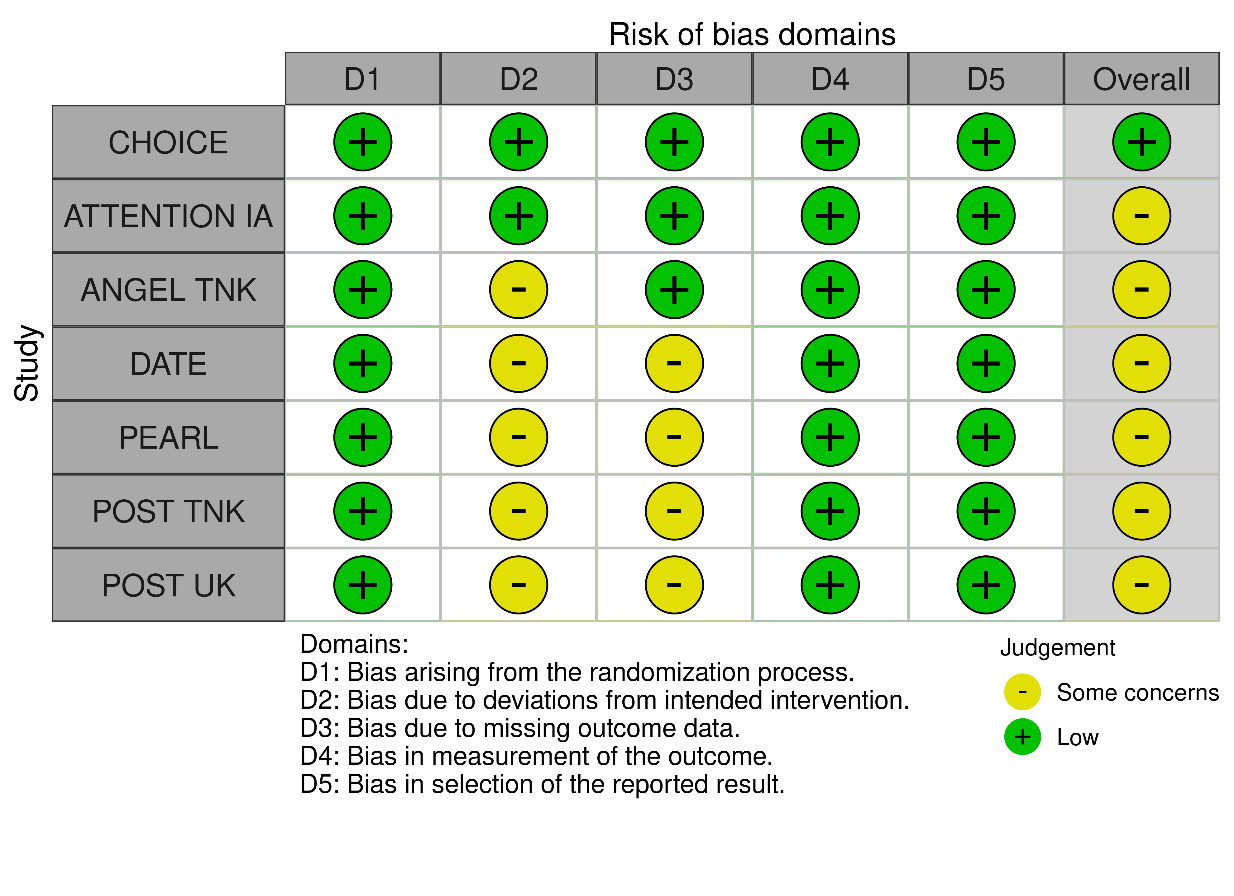


**B.**


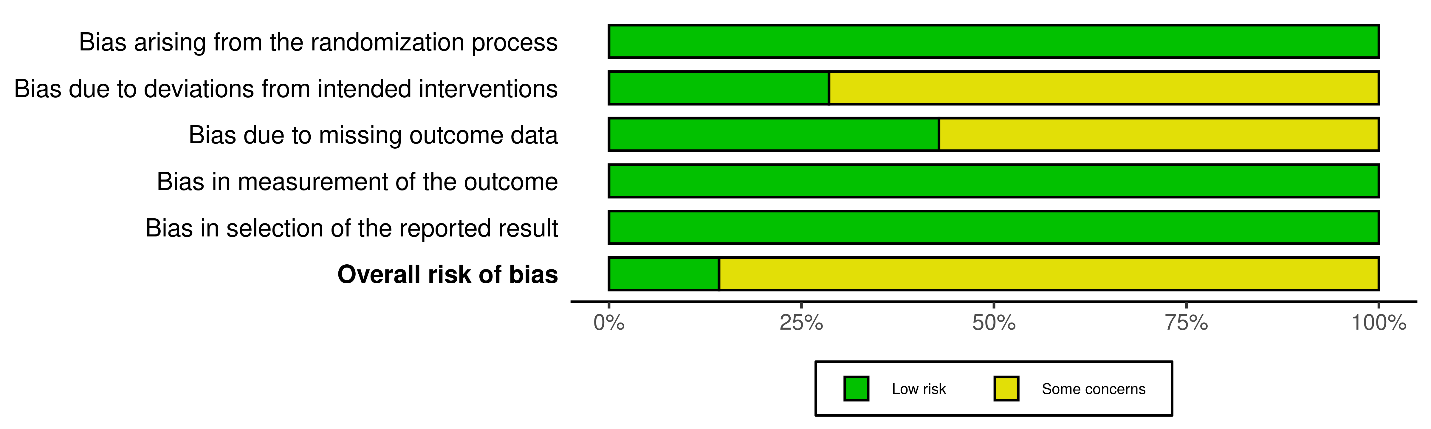


**eFigure 6.** Forest plot presenting the risk ratio of excellent functional outcome at 3 months among patients receiving intra-arterial thrombolysis (IAT) versus best medical treatment alone (BMT), stratified by country of recruitment in each trial.


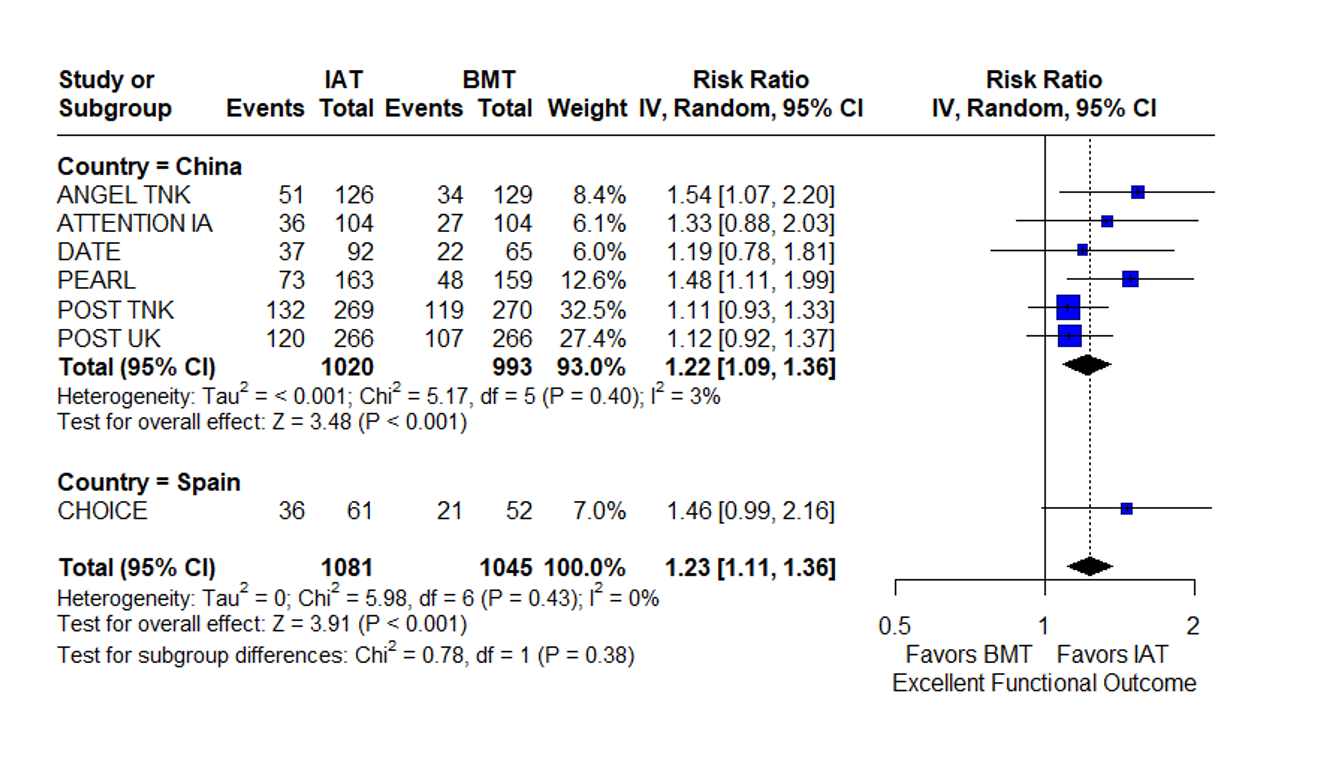


**eFigure 7.** Forest plot presenting the risk ratio of excellent functional outcome at 3 months among patients receiving intra-arterial thrombolysis (IAT) versus best medical treatment alone (BMT), stratified by pretreatment with intravenous thrombolysis (IVT; Panel A), relevant dose of thrombolytic drug (% licensed full-dose for intravenous thrombolysis; Panel B), or degree of reperfusion (eTICI 2b vs. 2c/3; Panel C).

**A.**


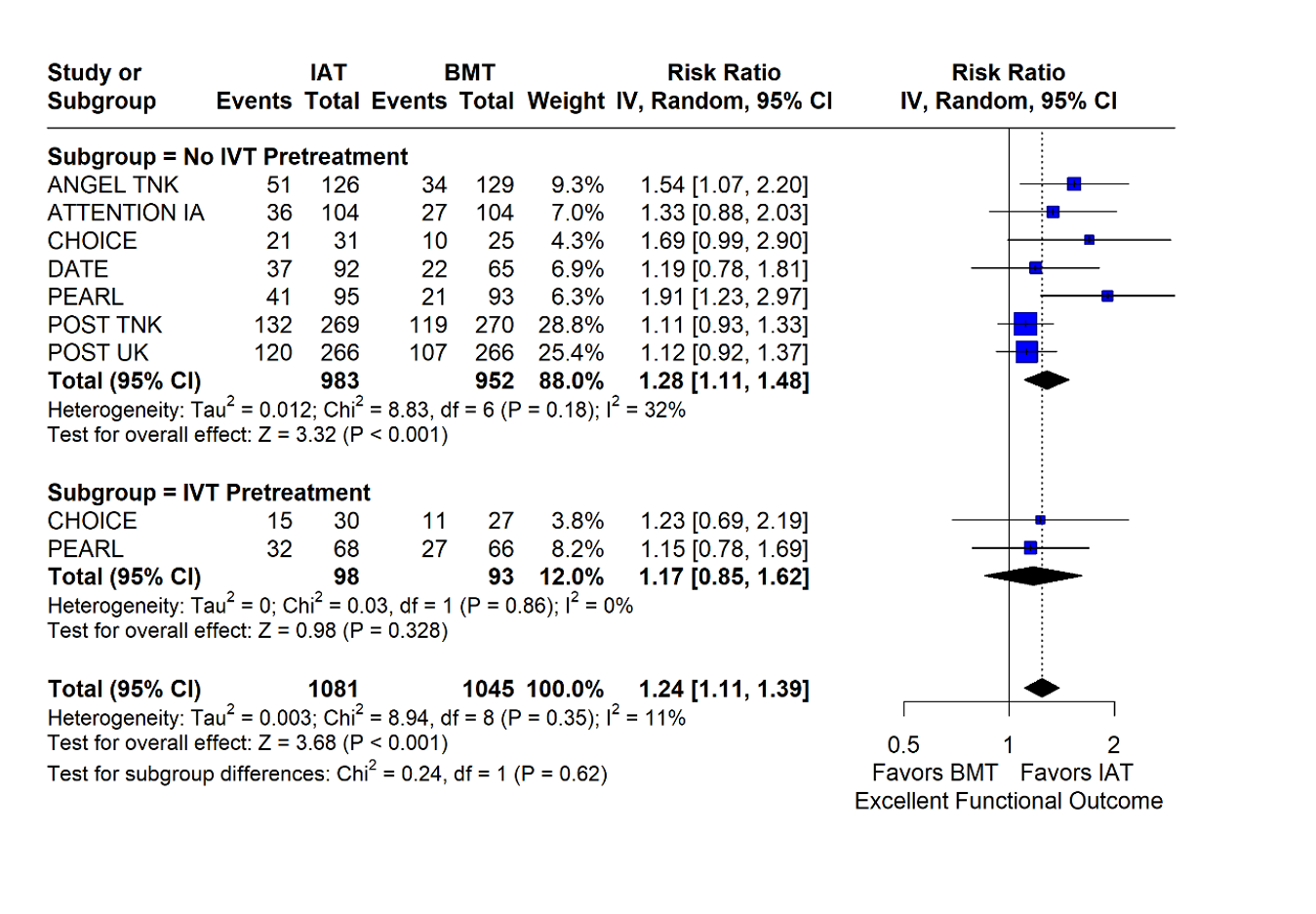


**B.**

**
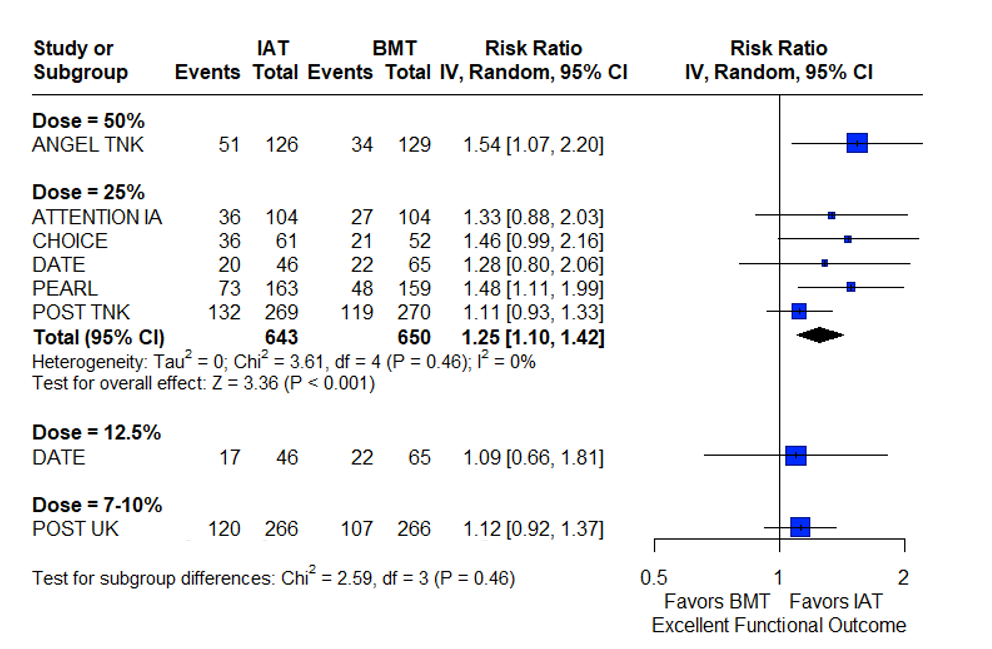
**

Note: The control group was used twice for the different doses of DATE trial, therefore, no overall result is provided.

**C.**

**
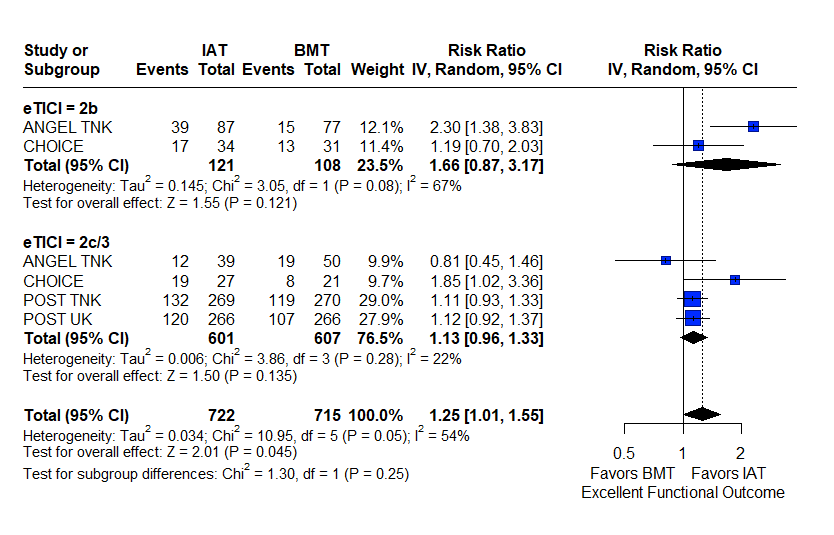
**

**eFigure 8.** Forest plot presenting the risk ratio of good functional outcome at 3 months among patients receiving intra-arterial thrombolysis (IAT) versus best medical treatment alone (BMT), stratified by the thrombolytic agent used.


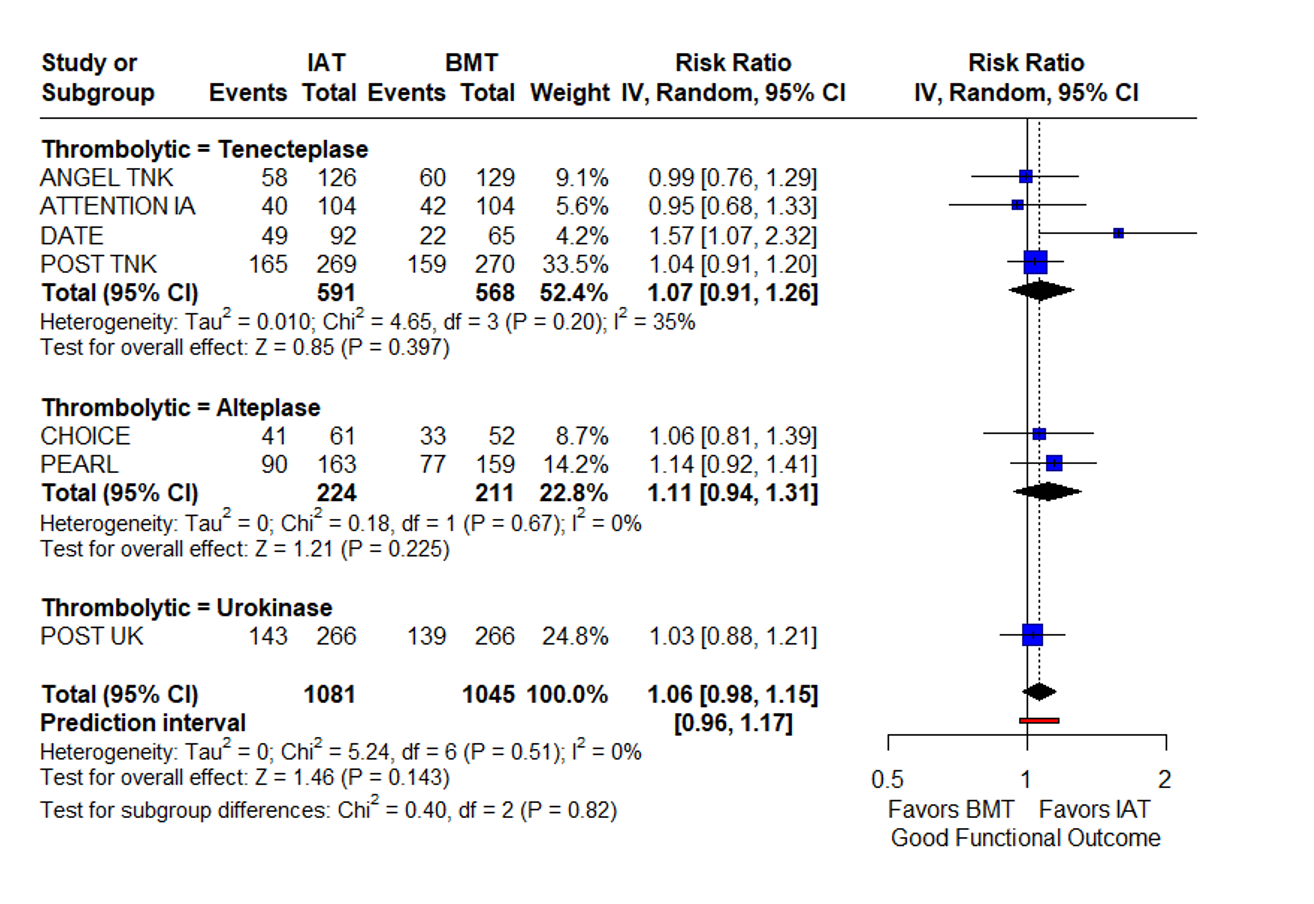


**eFigure 9.** Forest plot presenting the risk ratio of any intracranial hemorrhage among patients receiving intra-arterial thrombolysis (IAT) versus best medical treatment alone (BMT), stratified by the thrombolytic agent used.


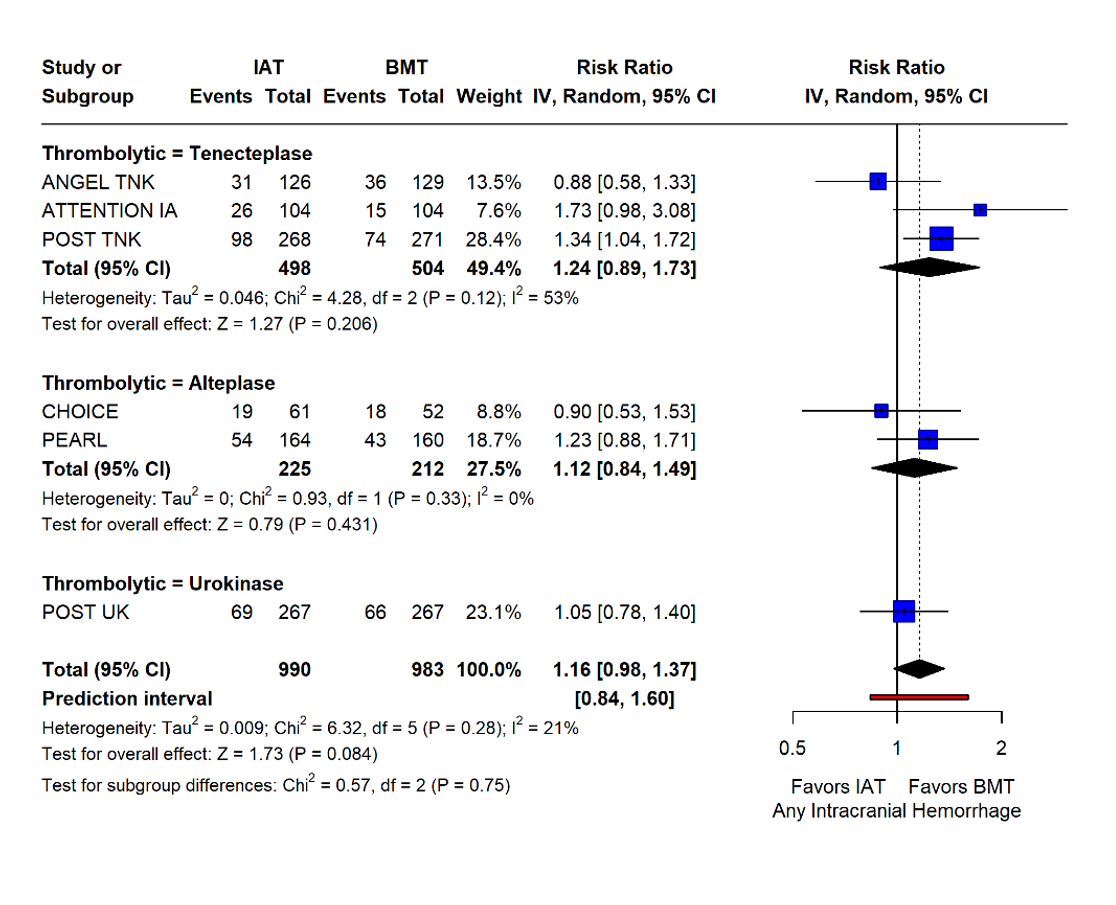


**eFigure 10.** Forest plot presenting the risk ratio of 3-month all-cause mortality among patients receiving intra-arterial thrombolysis (IAT) versus best medical treatment alone (BMT), stratified by the thrombolytic agent used.


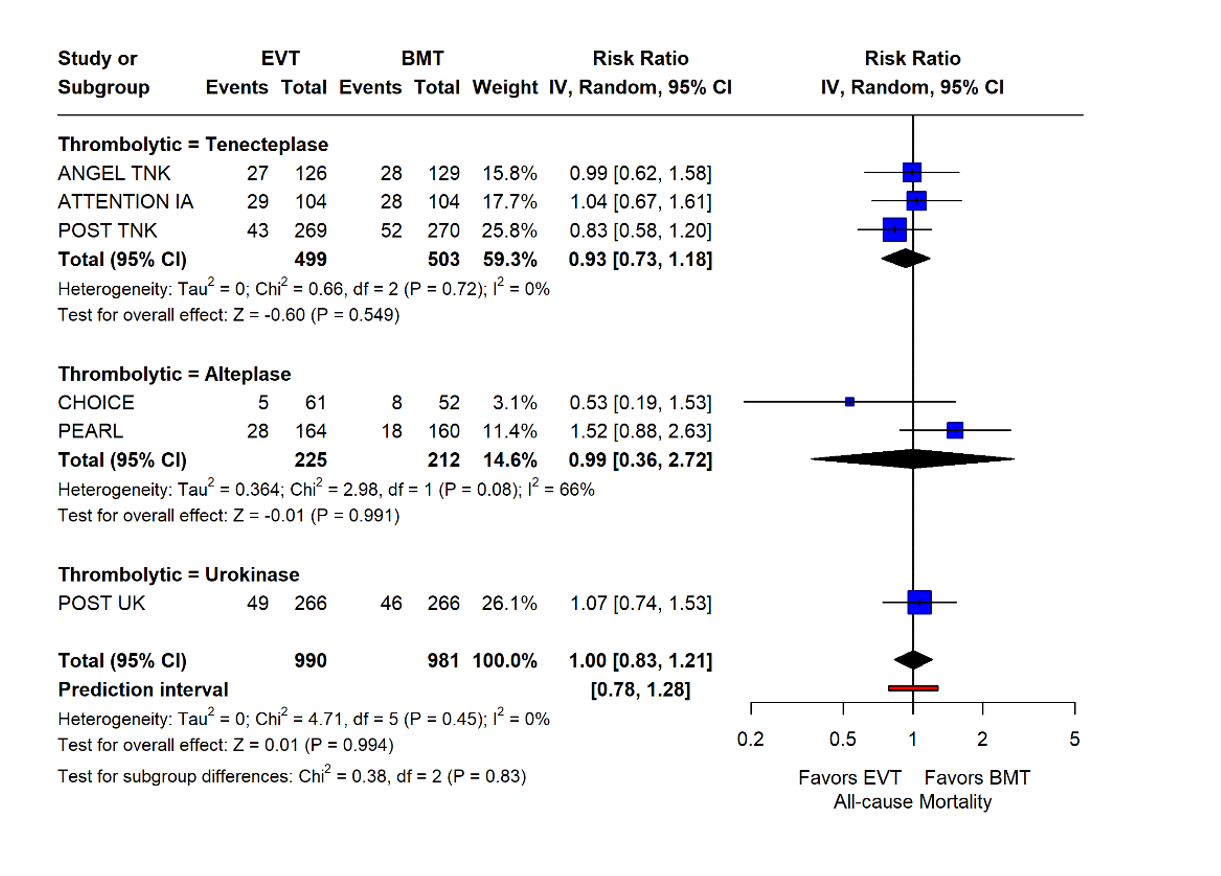


**eFigure 11.** Funnel plot on the reported rates of excellent functional outcome at 3 months.


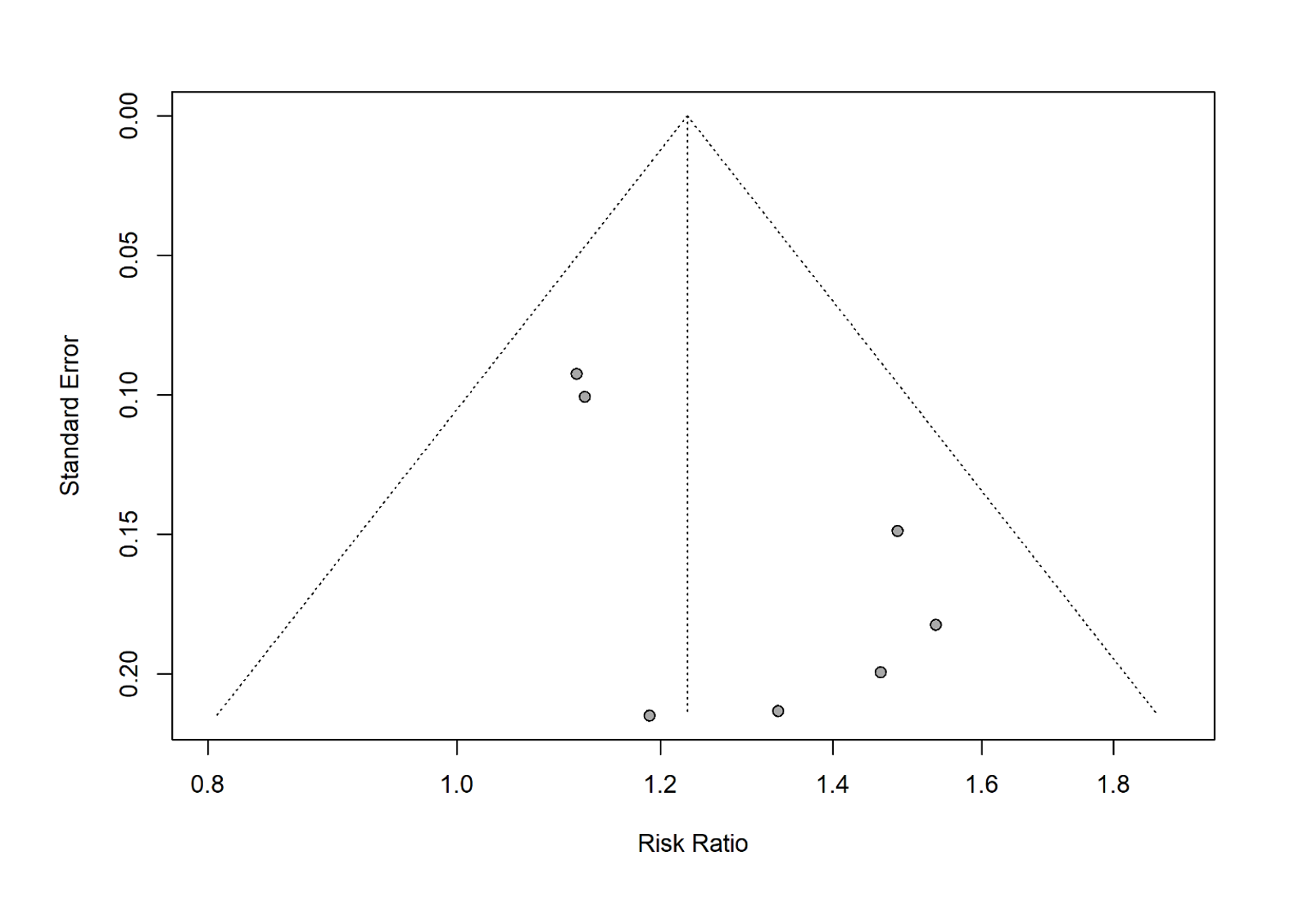


**eFigure 12.** Funnel plot on the reported rates of good functional outcome at 3 months.


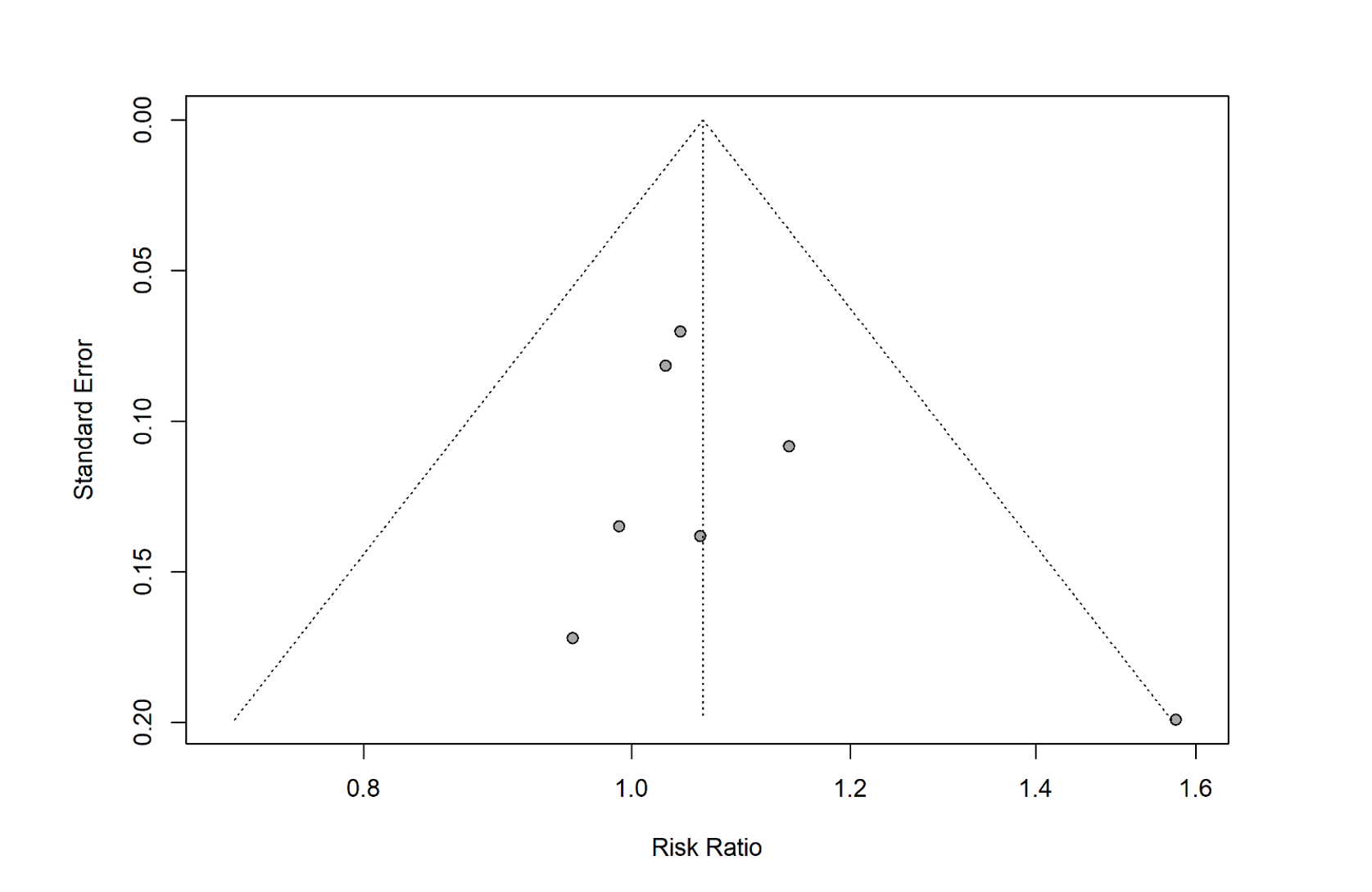


**eFigure 13.** Funnel plot on the reported odds of reduced disability at 3 months.


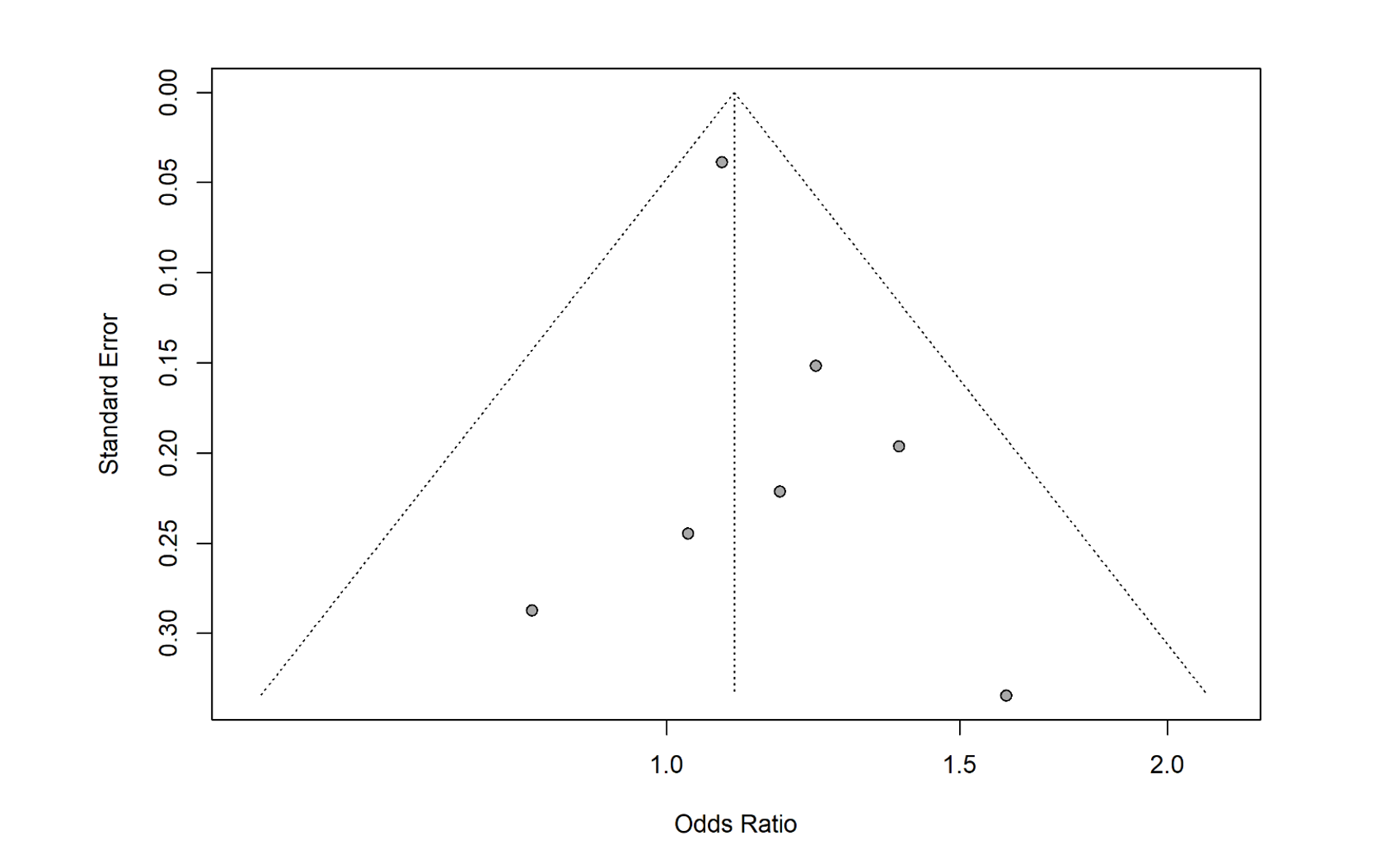


**eFigure 14.** Funnel plot on the reported rates of symptomatic intracranial hemorrhage.


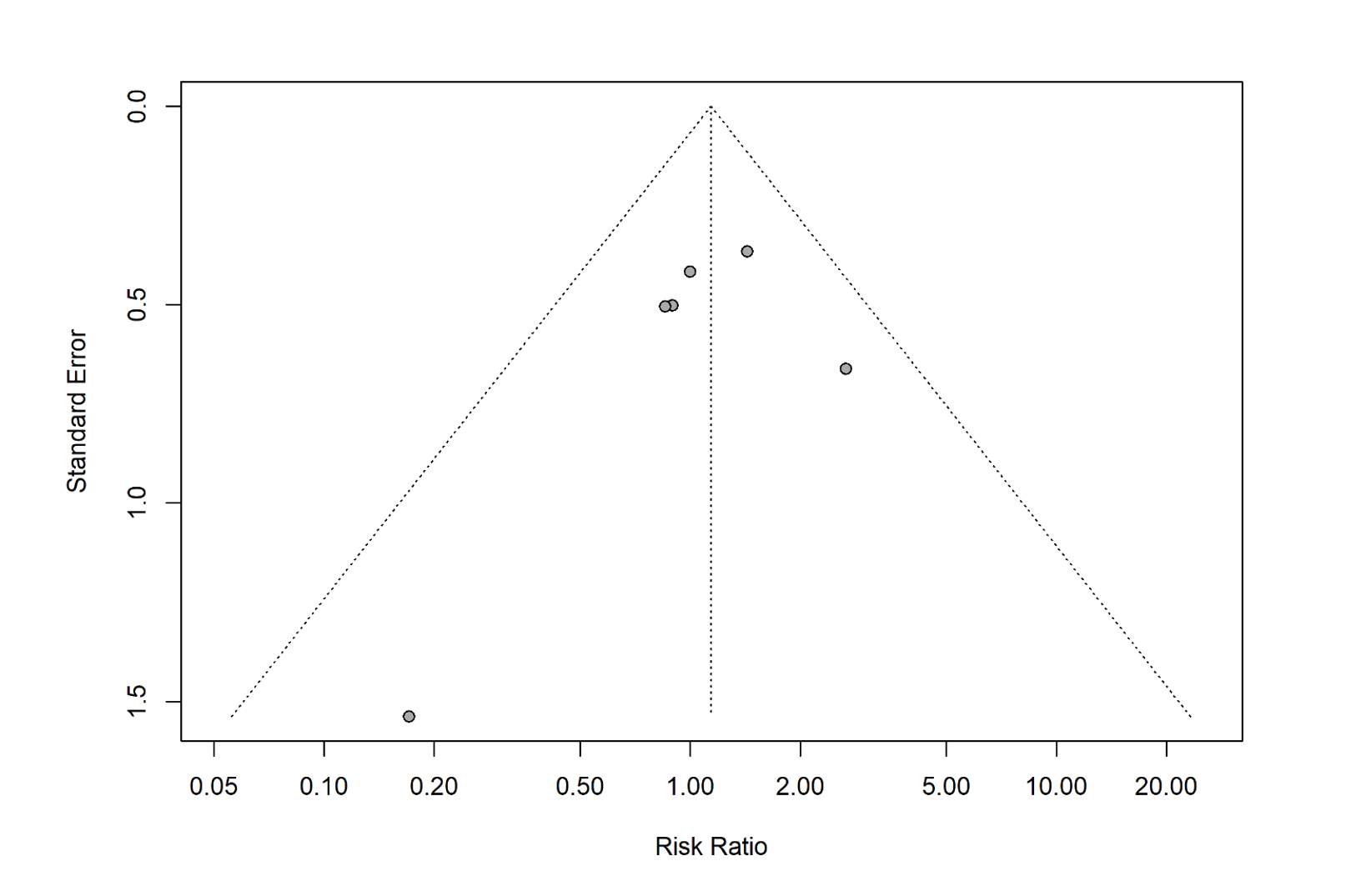


**eFigure 15.** Funnel plot on the reported rates of any intracranial hemorrhage.


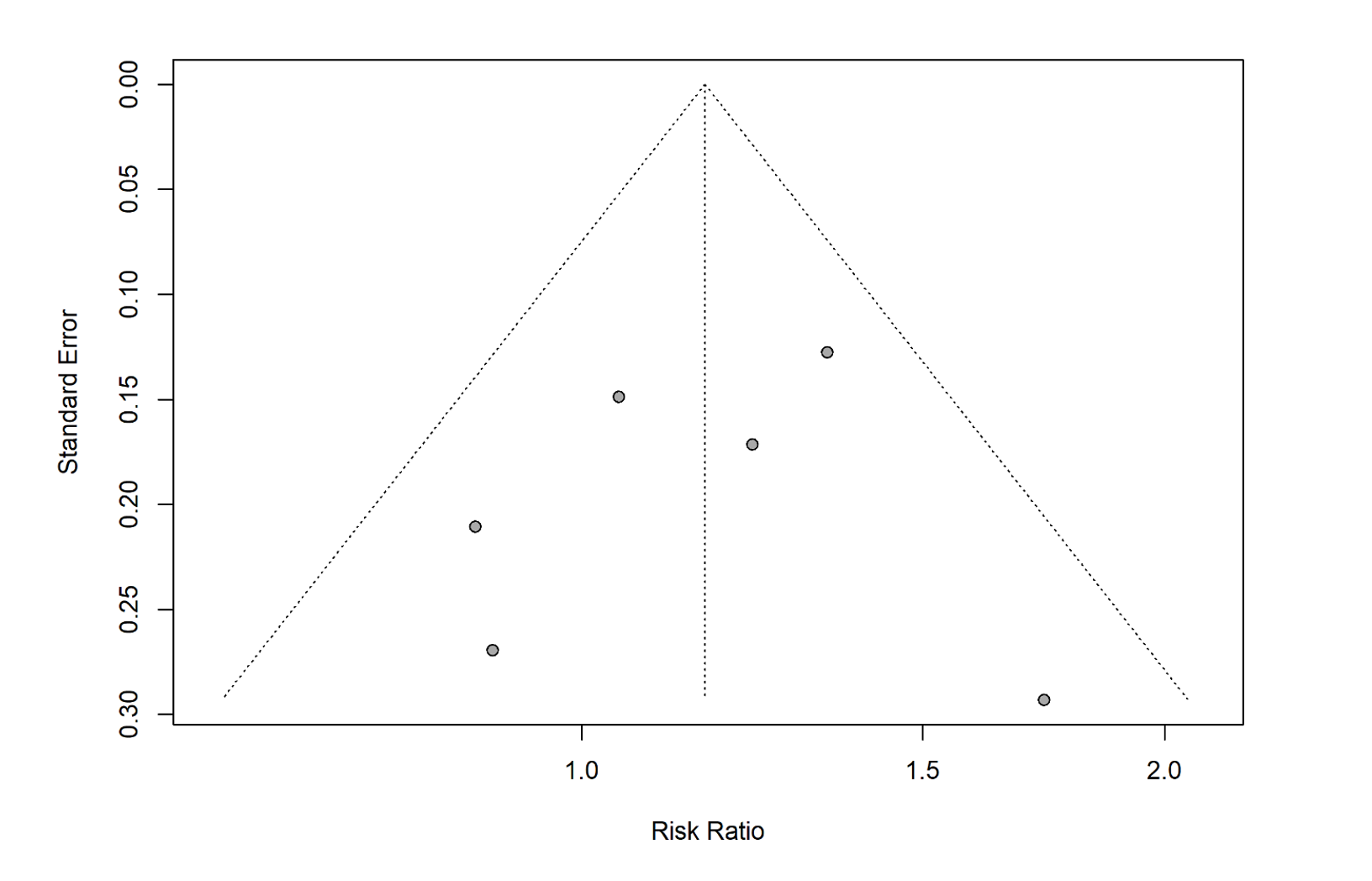


**eFigure 16.** Funnel plot on the reported rates of all-cause mortality at 3 months.


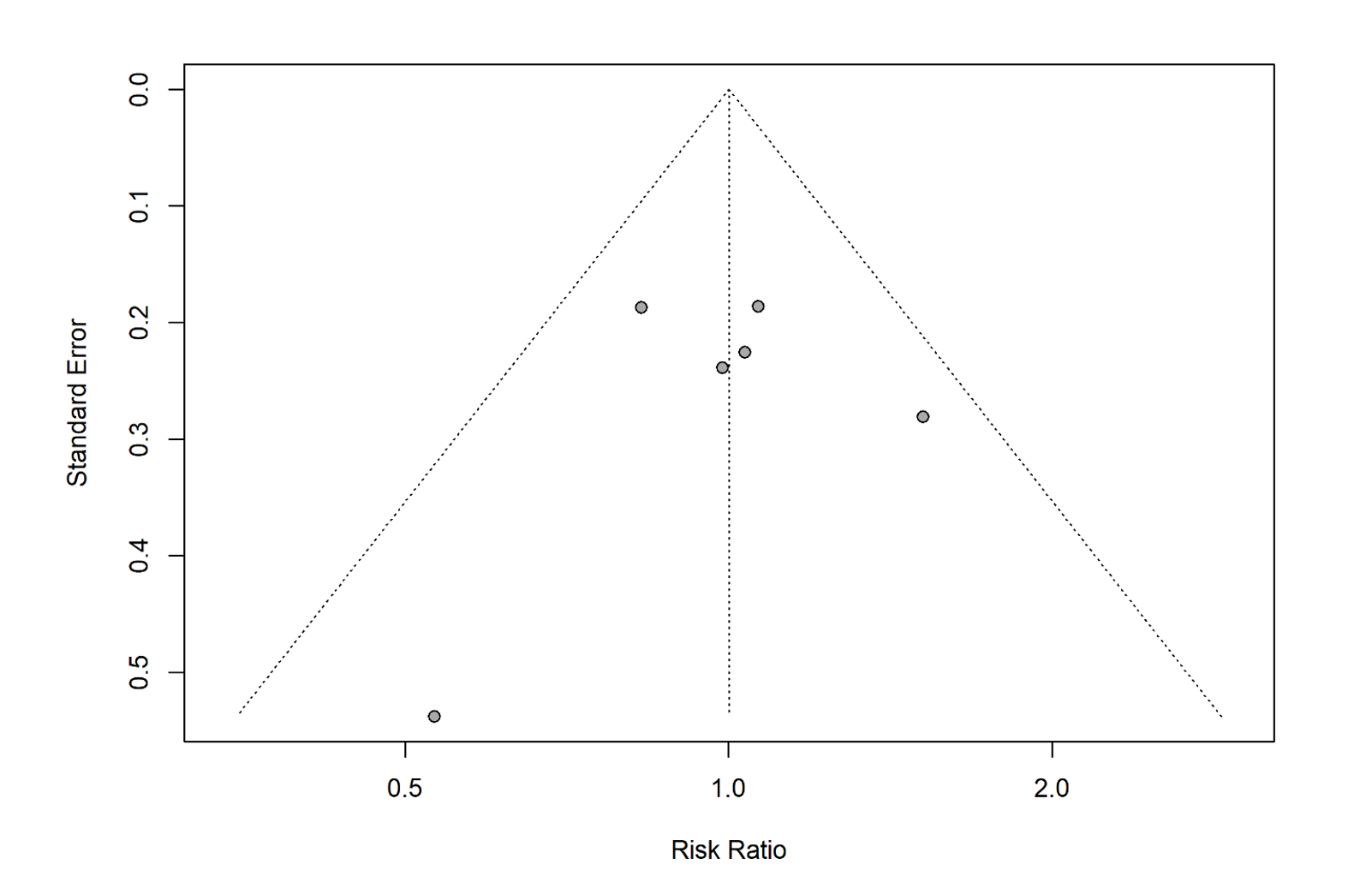

Supplement: Supplementary file 1 — Data S1. [file ENE-32-e70270-s001.docx]
